# Transcriptomic characterization of tissues from patients and subsequent pathway analyses reveal biological pathways that are implicated in spastic ataxia

**DOI:** 10.1186/s13578-022-00754-1

**Published:** 2022-03-11

**Authors:** Andrea C. Kakouri, Christina Votsi, Anastasis Oulas, Paschalis Nicolaou, Massimo Aureli, Giulia Lunghi, Maura Samarani, Giacomo M. Compagnoni, Sabrina Salani, Alessio Di Fonzo, Thalis Christophides, George A. Tanteles, Eleni Zamba-Papanicolaou, Marios Pantzaris, George M. Spyrou, Kyproula Christodoulou

**Affiliations:** 1 Department of Neurogenetics, The Cyprus Institute of Neurology and Genetics, 2370 Nicosia, Cyprus; 2 Department of Bioinformatics, The Cyprus Institute of Neurology and Genetics, 2370 Nicosia, Cyprus; 3 The Cyprus School of Molecular Medicine, The Cyprus Institute of Neurology and Genetics, 2370 Nicosia, Cyprus; 4 Department of Medical Biotechnology and Translational Medicine, University of Milan, 20090 Milano, Italy; 5 Unité de Trafic Membranaire ét PathogénèseDépartement de Biologie Cellulaire et Infection, Institut Pasteur, 75015 Paris, France; 6 Neurology Unit, Foundation IRCCS Ca’ Granda Ospedale Maggiore Policlinico, 20122 Milan, Italy; 7 School of Medicine and Surgery, University of Milano-Bicocca, 20126 Monza, Milan Italy; 8 General Surgical Department, Nicosia General Hospital, 2029 Nicosia, Cyprus; 9 Department of Clinical Genetics and Genomics, The Cyprus Institute of Neurology and Genetics, 2370 Nicosia, Cyprus; 10 Neurology Clinic D, The Cyprus Institute of Neurology and Genetics, 2370 Nicosia, Cyprus; 11 Neurology Clinic C, The Cyprus Institute of Neurology and Genetics, 2370 Nicosia, Cyprus

**Keywords:** Spastic ataxia, RNA-Seq, Transcriptomics, Neurodegeneration, Neurodegenerative disease, Pathways, Functional, Gene expression, Differential gene expression

## Abstract

**Background:**

Spastic ataxias (SAs) encompass a group of rare and severe neurodegenerative diseases, characterized by an overlap between ataxia and spastic paraplegia clinical features. They have been associated with pathogenic variants in a number of genes, including *GBA2*. This gene codes for the non-lysososomal β-glucosylceramidase, which is involved in sphingolipid metabolism through its catalytic role in the degradation of glucosylceramide. However, the mechanism by which *GBA2* variants lead to the development of SA is still unclear.

**Methods:**

In this work, we perform next-generation RNA-sequencing (RNA-seq), in an attempt to discover differentially expressed genes (DEGs) in lymphoblastoid, fibroblast cell lines and induced pluripotent stem cell-derived neurons derived from patients with SA, homozygous for the *GBA2* c.1780G > C missense variant. We further exploit DEGs in pathway analyses in order to elucidate candidate molecular mechanisms that are implicated in the development of the *GBA2* gene-associated SA.

**Results:**

Our data reveal a total of 5217 genes with significantly altered expression between patient and control tested tissues. Furthermore, the most significant extracted pathways are presented and discussed for their possible role in the pathogenesis of the disease. Among them are the oxidative stress, neuroinflammation, sphingolipid signaling and metabolism, PI3K-Akt and MAPK signaling pathways.

**Conclusions:**

Overall, our work examines for the first time the transcriptome profiles of *GBA2*-associated SA patients and suggests pathways and pathway synergies that could possibly have a role in SA pathogenesis. Lastly, it provides a list of DEGs and pathways that could be further validated towards the discovery of disease biomarkers.

**Supplementary Information:**

The online version contains supplementary material available at 10.1186/s13578-022-00754-1.

## Background

Spastic ataxias (SAs) comprise a group of rare neurodegenerative diseases that are characterized by an overlap between ataxia and spastic paraplegia clinical features. Their main symptoms include spasticity and weakness in the limbs, as well as gait and balance impairment, which are often accompanied by additional neurological or extra-neurological features. These diseases are also characterized by an early age of onset, usually before the age of 20 years. The neuronal structures that are mainly degenerated are the cerebellum, the brainstem, the spinocerebellar tract and/or the sensory tracts of the spinal cord, as well as the corticospinal tracts [1, 2].

SA associated pathogenic variants have been identified in genes originally associated with hereditary cerebellar ataxia (HCA) or hereditary spastic paraplegia (HSP) forms, including the *SPG7, SACS, FXN, ATXN1, CYP27A1, CAPN1, SETX, SYNE1, CACNA1A, AFG3L2, GBA2* and other genes [1, 3–5]. We have previously reported a novel missense *GBA2* variant (c.1780G > C, [p.Asp594His]) in a Cypriot consanguineous family with SA [5]. The *GBA2* gene encodes the non-lysosomal glucosylceramidase (GBA2), an enzyme that participates in the sphingolipid metabolism. In particular, GBA2 catalyses the removal of glucose from glucosylceramide (GlcCer) and the hydrolysis of bile acid–3-O-β-glucosides (BG) [6].

However, the exact mechanism by which the variant *GBA2* leads to the development of SA still remains unclear. Our previous biochemical investigation in lymphoblastoid cell lines (LCLs) of patients diagnosed with the biallelic *GBA2* c.1780G > C missense variant, showed lack of GBA2 enzyme activity, accumulation of GlcCer and increased activity of the lysosomal glucosylceramidase (GBA) [7]. Other in-vitro studies have shown that the GBA2 protein expression and activity in COS-7 and HeLa cells transfected with the missense variant c.1780G > C [p.Asp594His] was decreased compared to the wild type (WT) [8]. In addition, evaluation of the effect of this missense variant (p.Asp594His) and a frameshift variant on protein localization in HeLa, SH-SY5Y, primary rat hippocampal cells and in U2OS cells, demonstrated localization throughout the cell similar to that of the WT GBA2. On the other hand, C-terminally truncated GBA2 variants translocated to mitochondria, led to mitochondrial fragmentation and abolishment of the mitochondrial transmembrane potential in U2OS cells, which was not observed for the GBA2 missense variant Asp594His or the frameshift variant M510Vfs*17 (c.1528_1529del) [9]; nevertheless the possibility that this variant may have another uncharacterized association to mitochondrial function and/or related processes cannot be excluded. Based on the above, we hypothesize that the pathogenesis of SA could be due to dysregulation of the sphingolipid metabolism and loss of its enzymatic activity in patient derived LCLs that has already been demonstrated. However, we cannot exclude the possibility that GBA2 might have additional unknown function(s) and therefore additional pathways may contribute to the disease pathogenesis either through a synergistic and/or interacting activity with the sphingolipid metabolism, or through a sphingolipid-independent manner.

In this study, we perform comparative transcriptome analysis on LCLs, fibroblast cell lines (FCLs) and induced pluripotent stem cell (iPSC)-derived neurons from patients with the *GBA2* c.1780G > C variant and control individuals, in order to find genes with altered expression that could be further analysed, aiming at the elucidation of candidate pathways possibly implicated in the development of the *GBA2*-associated SA. Since neurons are the primarily affected cells in SA, we initially focused on the results of iPSC-derived neurons and compared them to FCLs and LCLs, in order to investigate if there are common genes and/or pathways that might be of significance. Furthermore, due to the limited availability of iPSC-derived neurons (only from a single patient and a control) and due to the possible existence of tissue specific pathological mechanisms, we further evaluated the data derived from FCLs and LCLs for which we had additional samples. Our results demonstrate a number of DEGs that clearly separate patient from control samples, some of which present with similar expression changes across the different tissues. We also highlight the most enriched pathways that result from the analyses performed. We finally report common candidate pathways to those that resulted from our previous work on the analysis of multiple gene expression datasets from ataxia and spasticity phenotypes [10], thus further supporting the implication of the hereby proposed pathways in SA.

## Methods

**Fig. 1:**
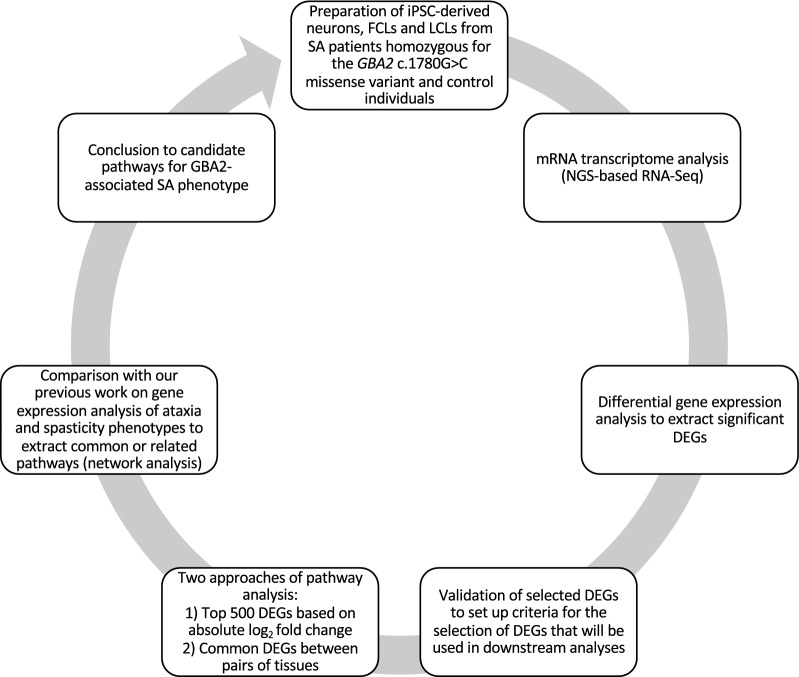
Flowchart of the study design

In this study, three types of cell lines from patients with SA and healthy individuals were expanded and used for transcriptome analysis in an attempt to identify genes that are differentially expressed in affected as compared to control individuals. Selected genes were further evaluated with an alternative quantitative method. The filtered lists of DEGs were then used as input in two different approaches of enrichment analysis for the discovery of candidate pathways that may be implicated in the development of SA. A schematic representation of our methodology is illustrated in Fig. 1.

### Cell cultures

#### Lymphoblastoid cell lines cultures

LCLs had been previously established using a standard B-cells Epstein-Barr virus (EBV) infection method for three patients with SA (PL1-3) and six non-SA control individuals (CL1-6), after informed consent. The cell lines were cultured and further expanded in RPMI 1640 medium (Biosera, Nuaille, France), supplemented with 10% FBS (ThermoFisher, Massachusetts, USA), and 10U/mL Penicillin/Streptomycin (Biosera).

#### Fibroblast cell lines cultures

FCLs were established from skin biopsies of two patients with SA (PF1-2), after informed consent. Sex and age matched adult human primary dermal fibroblasts were purchased from Lonza in order to be used as non-SA controls (CF1-2). The cell lines were further cultured and expanded in DMEM medium (GIBCO, Thermo-Fisher), supplemented with 10% FBS (Thermo-Fisher), and 10U/mL Penicillin/Streptomycin (Biosera).

#### iPSC-derived neurons

iPSCs from a patient and a non-SA control were generated from skin fibroblasts through a non-integrating reprogramming method based on the expression of factors *OCT4*, *SOX2*, *KLF4*, and *C-MYC*. Different clones of iPSCs were isolated and expanded [11]. The karyotype of all the lines was assessed to exclude genetic rearrangements due to the reprogramming process (data not shown). Dual SMAD inhibition was used for iPSCs’ differentiation. The protocol by Kriks et al. was modified in order to remove the differentiation factors which are specific for dopaminergic induction. The rationale of this approach to generate dorsal forebrain neurons is detailed in the paper itself [12]. Briefly, cells were cultured in proper media supplemented with specific factors at proper concentrations as follows. Day 0, 1, 2, 3, 4: KSR differentiation medium (81% DMEM, 15% KSR, 100X 1% non-essential amino acids, 100X 1%b-mercaptoethanol, 100X 1% penicillin/streptomycin, 100X 1% amphotericin) supplemented with 10 mM SB431542 and 100 nM LDN-193189. Days 5 and 6: 75% KSR differentiation medium and 25% N2 differentiation medium (97% DMEM, 100X 1% N2 supplement, 100X 1% penicillin/streptomycin, 100X 1% amphotericin) supplemented with 100 nM LDN-193189. Days 7 and 8: 50% KSR differentiation medium and 50% N2 differentiation medium supplemented with 100 nM LDN-193189. Days 9 and 10: 25% KSR differentiation medium and 75% N2 differentiation medium supplemented with 100 nM LDN-193189. From day 11 to the end of the differentiation: B27 differentiation medium (95% neurobasal medium, 50X 2% B27 supplement, 1% Glutamax, 100X 1% penicillin/streptomycin, 100X 1% amphotericin) supplemented with 10 ng/mL brain-derived neurotrophic factor (BDNF), 10 ng/mL glial cell line-derived neurotrophic factor (GDNF), 0.2 mM ascorbic acid, and 0.1 mM cyclic AMP. Morphological characterization using bright-field microscopy and expression of neuronal (TUJ1) and dopaminergic (TH) markers were used to demonstrate dopaminergic activity of the cells (Additional file 1: Fig. S1).

### RNA isolation

Total RNA was isolated from all the above cell lines, using the NucleoSpin RNA kit (MACHEREY–NAGEL, Germany), according to manufacturer’s instructions.

### cDNA library construction and sequencing

The Illumina TruSeq Stranded mRNA sample preparation Kit targeting poly-adenylated RNA (Illumina, San Diego, CA), was used for the cDNA library construction. An initial amount of 1 μg of total RNA was used for mRNA purification using poly-T oligo attached magnetic beads. Purified mRNA was then fragmented into small pieces using divalent cations under heating. The cleaved mRNA fragments served as templates for first strand cDNA synthesis followed by strand specificity marking and second strand cDNA synthesis, according to the manufacturer’s instructions. The final cDNA library of each sample was constructed through end-repair, adaptor-ligation, product purification and PCR enrichment. The cDNA libraries were qualified and quantified using TapeStation 2200 (Agilent, California, USA) and Qubit 2.0 Fluorometer (Invitrogen, California, USA). Qualified cDNA libraries were pooled and paired-end sequenced on our in-house Illumina NextSeq500 platform.

The following samples were initially used for this analysis: The three patient and three control LCL samples for a total of one technical replicate each, the two patient and the two controls FCL samples, the single patient and single control iPSC-derived neuron samples for a total of one technical replicate each.

A second experiment was performed in order to enrich the number of technical replicates for the tissues with limited biological replicate availability. cDNA library construction and sequencing were repeated for: the two patient and control FCL samples twice, thus having a total of three replicates per sample and for the patient and control iPSC-derived neuron samples once, thus having a total of two replicates per sample. All these second experiment produced libraries were single-end sequenced on the above in-house platform.

### Pre-processing of RNA-Seq data

Raw data were obtained in fastq format and quality control (QC) was performed using the FastQC tool version 0.11.5 [13] in command line. The Phred quality score was evaluated across reads and the raw sequences were then processed for adaptor filtering using the fastx-trimmer command of the FASTX-Toolkit version 0.0.14 [14] in command line. Mapping of raw reads was performed against the Genome Reference Consortium GRCh37 using TopHat version 2.1.1 [15]. The human reference genome was downloaded from the UCSC database in July 2019 [16]. FM indexing of the reference genome was performed prior to mapping using the Bowtie2 version 2.2.9 [17]. Quantification of mapped reads was performed using the HTSEQ-count tool [18].

### Differential expression analysis

Differential expression (DE) analysis was performed using the EdgeR package version 3.8 of R Bioconductor [19] for the identification of DEGs between patient and control samples. The gene counts were normalised for RNA composition between libraries using a trimmed mean of M-values (TMM) normalisation [20]. Genes with a minimum requirement of one count per million (CPM) across two or more libraries for each group (patient and control) have been kept. The QL F-test [20] was used as a statistical method to calculate the DEGs provided by the EdgeR package. Comparisons were made between patient and control samples for each of the three tissues analysed. The DEGs were calculated and a filter requirement was used to keep only the genes with a minimum of one CPM in at least two samples. Due to the small sample size, filtering by False-Discovery Rate (FDR) value failed to give significant DEGs in iPSC-derived neurons and FCLs. Therefore, a cut-off of p-value = 0.05 and absolute log_2_FC > 1 was used in order to extract significant results. Volcano plots and heatmaps were generated to show the proportion of significant over- and under-expressed genes and the pattern of expression of the top DEGs in SA patients and controls respectively. Hierarchical clustering was performed to group together: (a) similar samples and (b) similar genes based on the expression profiles.

### Quantitative real time-PCR

All RNA samples that were used for the RNA-seq analysis, as well as three additional LCL derived control samples have been used for RNA-seq results validation experiments, with qPCR. A range of 0.5–1 μg of total RNA were reverse transcribed to cDNA using the ProtoScript First Strand cDNA Synthesis Kit (New England BioLabs, Ipswich, MA, USA) and then were loaded on TaqMan Array Cards (which are ideal for achieving highly reproducible and sensitive results), (ThermoFisher) according to manufacturer’s instructions. Each card was comprised of eight channels of 48 individual reactions, in order to provide three technical replicates per sample for a total of eight samples and 16 tested genes per card. Predesigned and pre-validated TaqMan gene expression assays have been selected for the following genes: *MME, MTRNR2L1, HERC5, NTN1, PARP14, PLSCR1, SERINC5, PLD1, NR4A3, CYP7B1, ADAM23, STK17B, GLDC*, *SPG7*, *GAPDH* and *B2M*. The two latter housekeeping genes were used as endogenous controls. Cards were run on the QuantStudio 7 Flex Instrument (Applied Biosystems, California, USA). Relative quantification values were obtained using the QuantStudio software and the ΔΔC_T_ method. Based on the number of available samples, statistical analysis was indicated only for the case of LCLs (3 patients and 6 controls) derived data. The parametric two-sample t-test or the non-parametric Mann–Whitney U test have been used in R based on whether the data followed a normal distribution or not.

### Pathway enrichment analysis on the top 500 DEGs

Enrichment analyses were carried out using the DE analysis derived data. Filtering was performed in order to exclude any entries with no gene symbol, as well as any non-coding RNA with polyadenylation entries, from the DEGs lists. The top 500 DEGs of each tissue based on the highest absolute log_2_FC (p-value < 0.05) were used as input for the enrichment analysis, using the KEGG 2019 and Reactome 2016 pathway databases through the EnrichR tool [21]. The minimum absolute log_2_FC of the DEGs included in this analysis had been 2.098 indicating a high level of differentiation. A p-value of < 0.05 has been also finally used in order to extract significant pathway findings.

### Pathway enrichment analysis on the common DEGs between different tissues

A second round of pathway enrichment analysis was performed on the lists of common DEGs with p-value < 0.05 and an absolute log_2_FC > 1.2 (a criterion established after performing validation experiments as described below) between all tissues in pairs, using the KEGG 2019 and Reactome 2016 pathway databases through the EnrichR web-server. The derived pathways with p-value < 0.05 were considered as significant and have been sorted by their combined score. At this point we note that, in our view, the analysis based on the common DEGs between all the three tissues was not meaningful since they included only five protein coding genes.

### Pathway to pathway network construction

The results of pathway enrichment analysis of the top 500 DEGs for each tissue were compared to the pathways identified through our previous work on gene expression data analysis derived from ataxia and spasticity phenotypes-related microarray datasets [10]. We compared the pathways from the two studies based on the tissue similarity (i.e. pathways of iPSC-derived neurons from the current RNA-Seq analysis with the neuronal tissue pathways of the previous work, FCL-pathways of the current RNA-Seq analysis with FCL-pathways of the previous work). The pathways resulting from the RNA-Seq analysis of LCLs, were compared with the pathways resulting from peripheral blood datasets of our previous work, due to the limitation of finding enough LCL datasets to analyse. The intersection of pathways of the two studies was then used as seed pathways to construct pathway-to-pathway networks for each tissue using the PathwayConnector tool [21]. Extra pathways were added, using the shortest-path algorithm, in order to fill any missing connections between the pathway nodes or even to enrich already connected networks, for the identification of interesting pathway associations. In case there were more than one shortest paths with the same length, then all possible shortest paths were included into the final network.

## Results

RNA-seq transcriptome analysis was used to generate the molecular signature profile of samples from patients with SA and non-SA controls in an attempt to understand the mechanisms that are altered by the *GBA2* c.1780G > C pathogenic variant. The lists of DEGs were examined through bioinformatics tools, in order to investigate relevant pathways and biological processes that could be associated with the development of *GBA2*-related SA.

### Comparative analysis of transcriptome profiles in patients and controls

**Fig. 2:**
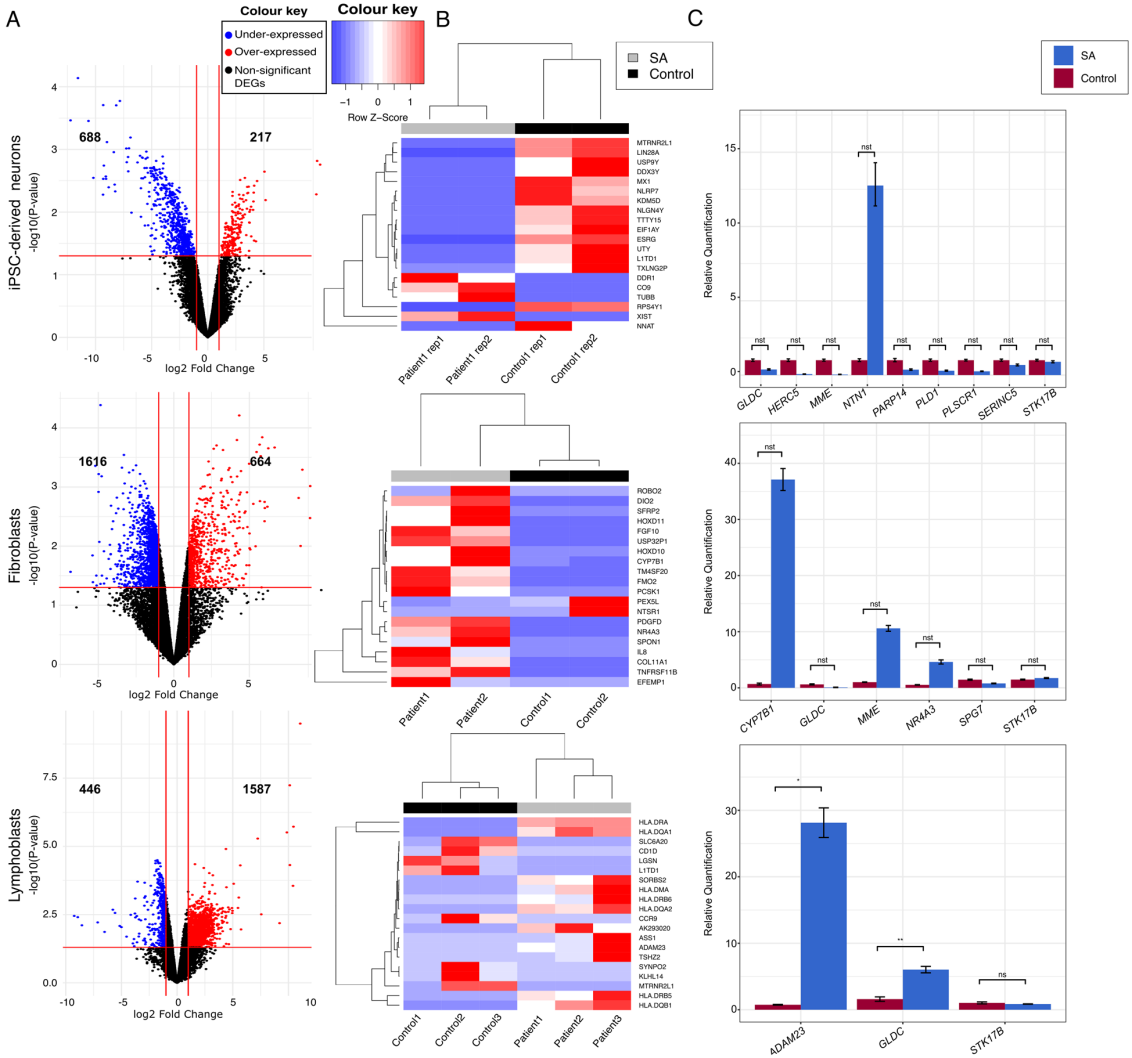
**A** Volcano plots of the calculated DEGs for each of the three tissues: iPSC-derived neurons, FCLs and LCLs. The number of over-expressed and under-expressed genes (absolute log_2_FC > 1) are indicated for p-value < 0.05. **B** Heatmaps of the top 20 DEGs based on absolute log_2_FC were generated for each tissue. Under-expressed genes are shown in blue and over-expressed genes in red. Hierarchical clustering was performed on samples and genes and a clear separation between patient and control is observed. **C** Validation of selected genes was performed using quantitative real-time PCR in patient and control samples of the three tissues. Statistical analysis was performed in LCL data, as it was the only tissue with 3 available biological replicates for each of the patient and control group, using Two sample t-test where data were normally distributed (*GLDC, STK17B*) and the Mann–Whitney U test, where data were non-normally distributed (*ADAM23*), as described in the methodology section. For iPSC-derived neurons and FCLs, statistical analysis was not performed due to the limited available sample size. The difference in the expression of genes between patients and controls in the two tissues is designated as non-statistically tested (nst). *nst*  non-statistically tested, *ns* non-significant, *p < 0.05, **p < 0.01, The DEGs with p-value < 0.05 are designated with *

A total number of 20–40 million reads was produced per sample with an average percentage mapping of 72% (Additional file 2). The raw count and normalized count per million (CPM) matrices are provided in Additional file 3. Differential expression analysis of the transcriptome profiles based on a threshold value of abslog_2_FC > 1 and p-value < 0.05 as significance criterion revealed: (a) 905 DEGs in iPSC-derived neurons of which 217 (24.0%) were over-expressed and 688 (76.0%) under-expressed, (b) 2280 DEGs in FCLs of which 664 (29.1%) were over-expressed and 1616 (70.9%) under-expressed and (c) 2033 DEGs in LCLs of which 1587 (78.1%) were over-expressed and 446 (21.9%) under-expressed. The top 20 DEGs (p-value < 0.05 and abslog_2_FC > 1) per tissue are presented in Fig. 2 and the full lists are presented in Additional file 4.

### Validation of selected genes

Fourteen DEGs *(MME, HERC5, NTN1, PARP14, PLSCR1, SERINC5, PLD1, NR3A3, CYP7B1, ADAM23, STK17B, GLDC, SPG7* and *MTRNR2L1)* and two housekeeping genes (*GAPDH* and *B2M)*, were selected for RNA-Seq results validation using quantitative Real time PCR assays (Fig. 2C). The majority of selected genes included iPSC-derived neurons extracted DEGs (10/14), as this tissue has been considered to be the closest to the primarily affected tissues in SA. Few of these genes were also identified as DEGs in one (*MME, MTRNR2L1*) or both (*GLDC, STK17B*) of the other two tested tissues, as shown in Fig. 2C. Additional FCLs (*CYP7B1, NR4A3, SPG7*) or LCLs (*ADAM23*) specifically extracted DEGs had been also selected. Moreover, the selection covered a wide range (1.08–9.32) of absolute log_2_FC values all with p-value < 0.05 in all tissues, except for *STK17B* (only in FCLs and LCLs p-value < 0.05, Additional file 5) in order to establish a minimum threshold of absolute log_2_FC value to be used for the inclusion of DEGs in subsequent analyses. The reported biological role of the DEGs, had also been considered through this selection and those with any possible association to neurodegeneration or with known variants causing SA had been prioritized (Table 1).

**Table 1:** List of the genes that were selected for qRT-PCR validation

Gene symbol	Encoded protein	Protein description
*MME*	Neprilysin	A zinc-dependent metalloprotease/one of the most prominent beta amyloid (Aβ) degrading enzymes [22, 23]. Previous association with a number of neurological disorders, including Friedreich’s ataxia (FRDA), Charcot-Marie Tooth (CMT), autosomal recessive distal hereditary motor neuropathy and autosomal dominant spinocerebellar ataxia with neuropathy [22–24]
*HERC5*	E3 ISG15–protein ligase	Participates in mitophagy and autophagy regulation through its association with ISG15. Deregulation of these processes by ISG15 was described in ataxia telangiectasia and other neurodegenerative diseases [25, 26]. Altered mRNA expression of *HERC5* identified in iPSCs and neurons of a patient with Parkinson disease (PD) [27], as well as in skeletal muscle of Duchenne muscular dystrophy (DMD) patients [28]
*NTN1*	Netrin-1	Belongs to the family of laminin-related secreted proteins. Under-expressed mRNA in the brain of mucopolysaccharidosis type II mouse models [29] and up-regulated protein in brain tissue and cerebrospinal fluid of Alzheimer disease (AD) patients and mouse models [30] previously identified
*PARP14*	poly (ADP-ribose) polymerase family member 14	A known anti-apoptotic protein with possible role in the monitoring of aerobic respiration [31]
*PLSCR1*	Phospholipid scramblase 1	Involved in the reorganization of the phospholipid bilayer of the plasma membrane. Its activation might result to increased phosphatidylserine levels at the plasma membrane, which is indicative of apoptotic or energy-deprived cells and αβ toxicity. Also implicated in calcium homeostasis in neuronal cells, as well as autophagy, and associated with AD and cancer [32, 33]
*SERINC5*	Serine incorporator 5	Involved in myelination. It adds serine into newly forming membrane lipids and is enriched in myelin in the brain [34]
*PLD1*	Phospholipase D1	Involved in the regulation of cytoskeleton organization in neurons, in dendritic branching and spine regulation. Downregulation of *PLD1* has been described to affect α-synuclein-triggered neurodegeneration in PD [35]
*NR4A3*	Nuclear receptor subfamily 4, group A, member 3	Also known as neuron-derived orphan receptor-1. It is involved in various biological processes like the cell cycle, neuronal differentiation, apoptosis and metabolism. It can also act as a transcription factor. Its homolog NR4A2 has been previously linked to the pathogenesis of PD [36]
*CYP7B1*	Cytochrome P450 Family 7 Subfamily B Member 1	A protein of the cytochrome P450 superfamily of enzymes. *CYP7B1* variants have been reported in pure and complex forms of hereditary spastic paraplegia 5, as well as bile acid metabolism disorder [37, 38]. The enzyme has a role in cholesterol metabolism and primary bile acid production and it is widely expressed in the liver and brain [37]
*ADAM23*	ADAM Metallopeptidase 23	A cell-surface glycoprotein expressed in CNS neurons [39]. It participates in several biological processes, such as myoblast differentiation, growth factor secretion and others, and has been shown to be dysregulated in PBMCs from classic ataxia telangiectasia patients [39, 40]. Also shown to have altered expression in blood and brain of PD patients [41]
*STK17B*	Serine/Threonine Kinase 17b	Acts as a positive regulator of apoptosis [42]. It has been suggested to promote carcinogenesis and metastasis through its involvement in the AKT/GSK-3β/Snail signaling pathway [43]
*GLDC*	Glycine dehydrogenase	Catalyses the breakdown of glycine, that is involved in fatty acid response, and has a protein homodimerization activity. It has been shown to undergo methylation alterations in aging [44], while variants of the gene were reported in cases of corticobasal degeneration, progressive supranuclear palsy and frontotemporal dementia [45]
*SPG7*	Paraplegin	A mitochondrial metalloprotease with many reported variants causing SA, HSP or HCA. It disrupts mitochondrial dynamics and calcium homeostasis [46]. It is highly expressed in large neurons, such as cortical pyramidal neurons and Purkinje cells and associates with AFG3L2 to form a hetero-oligomeric complex. Variants of the *AFG3L2* gene have been previously reported to cause spinocerebellar ataxia type 28 and autosomal recessive SA type 5 [47]
*MTRNR2L1*	Humanin-like protein 1	Neuroprotective and anti-apoptotic role in cortical neurons [48]

All of the tested genes showed analogous expression change in our qPCR quantitation assays to that observed in the RNA-Seq experiments (Additional file 5), except for the *MTRNR2L1*, which failed to produce consistent results due to its minimal expression in all but one LCL control sample. Therefore, *MTRNR2L1* was excluded from further analysis. Statistical analysis was performed for *ADAM23*, *GLDC* and *STK17B* data derived from LCLs only, and confirmed the change in expression as significant for the first two but not for the *STK17B* (Fig. 2C). Through the RNA-Seq analysis, the *STK17B* gene had been found under-expressed but not significant in iPSC-derived neurons (log_2_FC = − 1.83, p-value = 0.076), significantly over-expressed in FCLs (log_2_FC = 1.03, p-value = 0.04) and significantly under-expressed in LCL patient cells (log_2_FC = − 1.17, p-value = 0.0013). A similar expression trend was observed through qPCR for each tissue but statistical analysis in LCLs did not confirm its significance. Our results suggest that the RNA-Seq derived DEGs with absolute log_2_FC >  ~ 1 and p-value < 0.05 show comparable expression changes in both RNA-Seq and qPCR. Thus, the statistical significance for all the tested genes with absolute log_2_FC > 1.2 was confirmed except for the *STK17B* gene. Even though such differences might be attributed to the small sample size and the different statistical methods used in the two approaches, in order to avoid the possibility of extracting any false findings, the absolute log_2_FC > 1.2 threshold value was set as a selection criterion for the subsequent pathway analyses performed.

### Hereditary cerebellar ataxia and hereditary spastic paraplegia associated genes showing differential expression in patients

SA is characterised by an overlap in clinical features of ataxia and spastic paraplegia. For this reason, we searched specifically for the presence of HCA and HSP known genes in the lists of the identified DEGs in the current study in order to indicate their possible involvement in *GBA2*-associated SA and therefore the existence of common pathway(s) with other SA, HCA or HSP type(s). Eight known genes were found to be differentially expressed in iPSC-derived neurons of SA patients, 27 in FCLs and 22 in LCLs (Additional file 6). *MARS*, *CAPN1*, *STUB1* and *CSTB* genes were common between FCLs and LCLs and the *SLC1A3* gene was common between FCLs and iPSC-derived neurons (Additional file 6). Although these have been considered as interesting findings, only *SLC1A3* showed an expression change in the same direction in both iPSC-derived neurons and FCLs, with log_2_FC values of 1.76 and 2.37 respectively.

*MARS* codes for the aminoacyl-tRNA synthetase catalysing the aminoacylation of tRNAs. It participates in the selenocompound metabolism and gene expression pathway. *CAPN1* codes for calpain 1, a calcium-activated neutral protease that catalyses the degradation of substrates involved in cytoskeletal remodelling. *STUB1* codes for E3 ubiquitin-protein ligase that participates in the TGF-β receptor signaling. *CSTB* codes for stefin, a member of the cystatin protein family, that acts as an intracellular thiol protease inhibitor and is associated to the innate immune system. *SLC1A3* codes for a member of the high affinity glutamate transporter family, which are proteins involved in the neurotransmission in the CNS. Among its associated pathways are the neurotransmitter release cycle, synaptic vesicle cycle, transport of glucose and other sugars, bile salts and organic acids, metal ions and amine compounds.

### Altered expression in more than one tissue of patients

A comparison of all the DEGs (p-value < 0.05, absolute log_2_FC > 1.2) between the three tissues, aiming at the discovery of genes with altered expression in more than one tissue of SA affected individuals, generated a list of common DEGs presented in Additional file 7. Six genes were found differentially expressed in all three tissues: *C1orf115, DOCK9, TMEM132B, CYB5R2, USP32P1* and *GLDC* (Table 2). *USP32P1,* being a pseudogene, was excluded from further downstream consideration.

**Table 2:** The five protein-coding genes that were found to be differentially-expressed in all three tissues of patients with SA (iPSC-derived neurons, FCLs and LCLs)

Gene name	Encoded protein	Description
*C1orf115*	Chromosome 1 open reading frame 115	Previously characterised to be down regulated in severe AD [49]. Also found under-expressed in Ataxia-telangiectasia cerebellar cortex [50]
*DOCK9*	Dedicator of cytokinesis protein 9	Member of DOCK proteins (atypical guanine nucleotide exchange factors-GEFs) associated with a broad range of neurodevelopmental, neuropsychiatric and neurodegenerative diseases, such as AD, PD, HD and ALS [51]). Involved in dendrite development and associated with bipolar disorder [52, 53]
*TMEM132B*	Transmembrane protein 132B	Variants in the genes of the TMEM132 family are associated with hearing loss, panic disorder or cancer [54]
*CYB5R2*	Cytochrome B5 Reductase 2	Participates in cholesterol biosynthesis and fatty acid desaturation and elongation [55, 56]. It is a plasma membrane redox enzyme, found in neuronal synaptic vesicles and its role is to control the redox state and bioenergetics for the protection of neuronal cells against metabolic and oxidative stress [57]. Reported to generate superoxide anion [58], which can in turn affect the onset of apoptosis of cultured cerebellar granule neurons (CGN) [59]
*GLDC*	Glycine dehydrogenase	Critical enzyme in glycine degradation. Variants of the *GLDC* gene cause glycine encephalopathy due to increased levels of glycine in the brain. GLDC can also affect the pattern of brain atrophy and the clinical features in tauopathies [60]

In addition to the above comparison including all the three tissues, tissue pairwise comparisons (using a p-value < 0.05 and an absolute log_2_FC > 1.2 as DEGs selection criteria) were performed towards the possible identification of interesting genes that had not been extracted through the first comparison: either due to the possible existence of tissue specific differential gene expression profile (including a limited or absent expression of some genes in one of the tissues), or the possible existence of tissue specific pathological mechanisms, or the limited number of available biological replicates. Pairwise comparison of iPSC-derived neurons and FCLs DEGs showed 84 common genes, including *SPON1*, which was over-expressed in patient cells of both tissues (log_2_FC = 2.89 and log_2_FC = 7.61 respectively—Additional file 7). However, no counts had been obtained in LCLs samples, indicating no expression in this tissue and justifying for its absence in the first comparison. *SPON1* encodes for the spondin 1 protein that has been previously associated with Alzheimer's disease (AD) and dementia [61]. Comparison of iPSC-derived neurons and LCLs revealed 43 common DEGs, whereas comparison of FCLs and LCLs revealed a total of 255 common DEGs. The lists of the common DEGs resulting from all the above comparisons are provided in Additional file 7.

### Pathway analysis of the top-scored DEGs

**Fig. 3:**
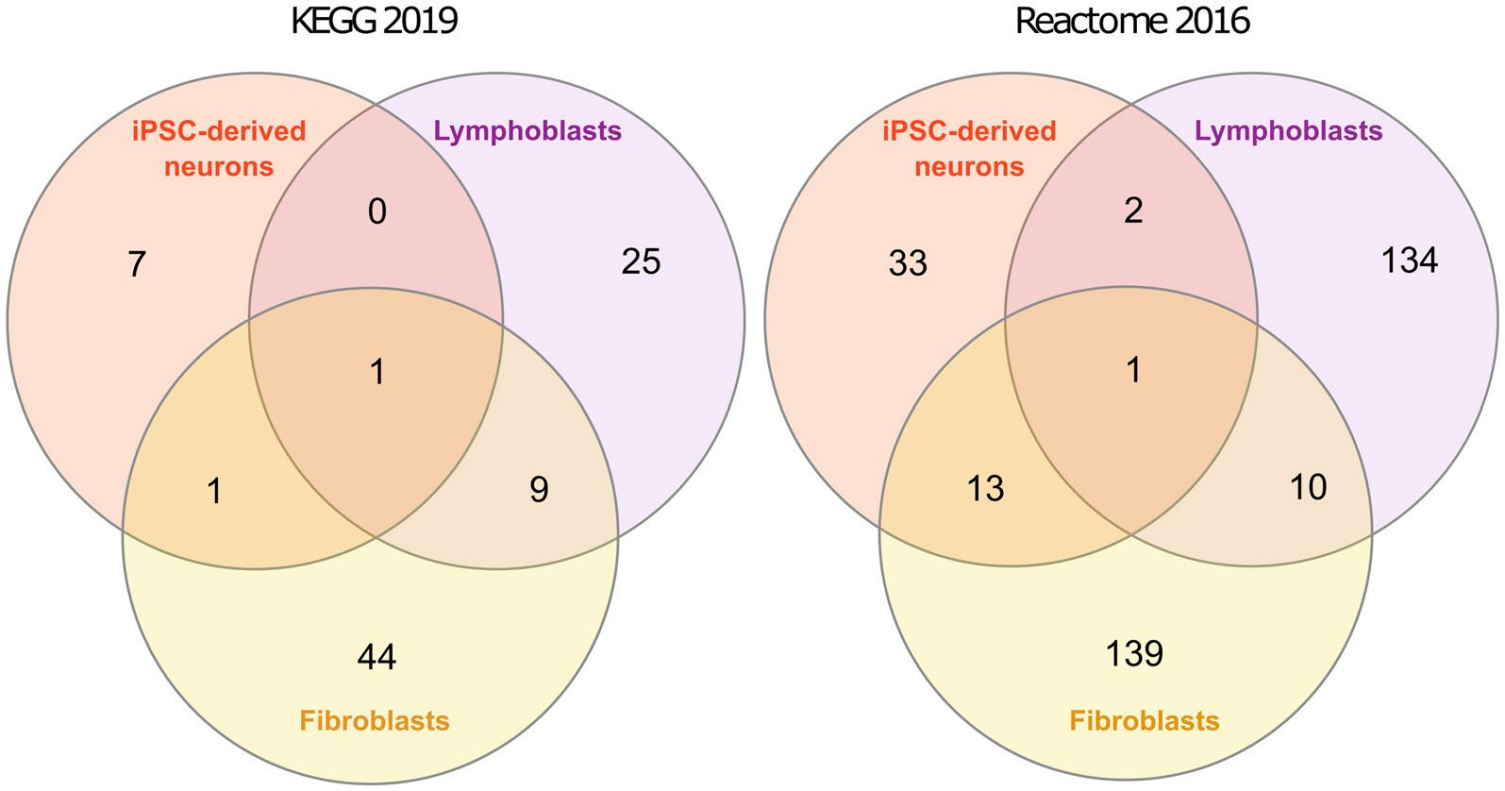
Venn diagrams presenting the number of common KEGG 2019 and Reactome 2016 pathways for each tissue analysed

In order to highlight the most enriched pathways for the disease under study, enrichment analysis was performed using the top 500 DEGs of each of the three tissues as described in the methodology section. The full lists of the identified pathways are presented in Additional file 8. Comparison of the KEGG 2019 and Reactome 2016 pathways across the different tissues (Table 3) has shown KEGG 2019 “Proteoglycans in cancer” and Reactome 2016 “Cytokine Signaling in Immune System” as the only common pathways between all three tissues. Venn diagrams summarizing the number of common pathways between the pairs of tissues are also presented in Fig. 3.

**Table 3:** KEGG 2019 and Reactome 2016 resulting pathways that were common between the three tissues

Database	iPSC-derived neurons and FCLs and LCLs	iPSC-derived neurons and FCLs	iPSC-derived neurons and LCLs	FCLs and LCLs
KEGG 2019	Proteoglycans in cancer	Melanoma		Salmonella infection
				Malaria
				C-type lectin receptor signaling pathway
				Chagas disease (American trypanosomiasis)
				Legionellosis
				Amoebiasis
				Hematopoietic cell lineage
				Cytokine-cytokine receptor interaction
				Axon guidance
Reactome 2016	Cytokine signaling in immune system	Defective CHST6 causes MCDC1	Interferon alpha/beta signaling	Termination of translesion DNA synthesis
		Defective ST3GAL3 causes MCT12 and EIEE15		DNA Damage Bypass
		Defective B4GALT1 causes B4GALT1-CDG (CDG-2d)		Gap-filling DNA repair synthesis and ligation in GG-NER
		Synthesis of Prostaglandins (PG) and Thromboxanes (TX)		Growth hormone receptor signaling
		Non-integrin membrane-ECM interactions		Nicotinate metabolism
		Keratan sulfate degradation		Signaling by Interleukins
		Extracellular matrix organization		Recognition of DNA damage by PCNA-containing replication complex
		Diseases associated with glycosaminoglycan metabolism	Immune system	Rho GTPase cycle
		HS-GAG biosynthesis		
		Glycosaminoglycan metabolism		Senescence-Associated Secretory Phenotype (SASP)
		Heparan sulfate/heparin (HS-GAG) metabolism		
		ECM proteoglycans		Axon guidance
		Cell surface interactions at the vascular wall

### Pathway analysis of the intersection of DEGs across tissues supports that the GBA2 c.1780G > C pathogenic variant affects important biological pathways

As the above pathway analysis revealed a limited number of common pathways between the tested tissues, another approach was employed aiming at the possible extraction of more common findings between the three or at least two of the tested tissues, as such type of data (confirmed by more samples) could be considered as more significant. In this analysis, only the common genes resulting from the DE analysis between the tissues in pairs were used, because the number of common genes between the three tissues was too small. Initially, we chose to focus on the common genes between the iPSC-derived neurons and FCLs or LCLs, as the first is the closest to the affected tissue in the studied disease. As mentioned above, due to the possible existence of tissue specific gene expression profile or pathological mechanisms, as well as due to the limited number of iPSC-derived neuron samples, we also evaluated the list of common DEGs between FCLs and LCLs, in order to discover pathways that would be otherwise missed.

**Fig. 4:**
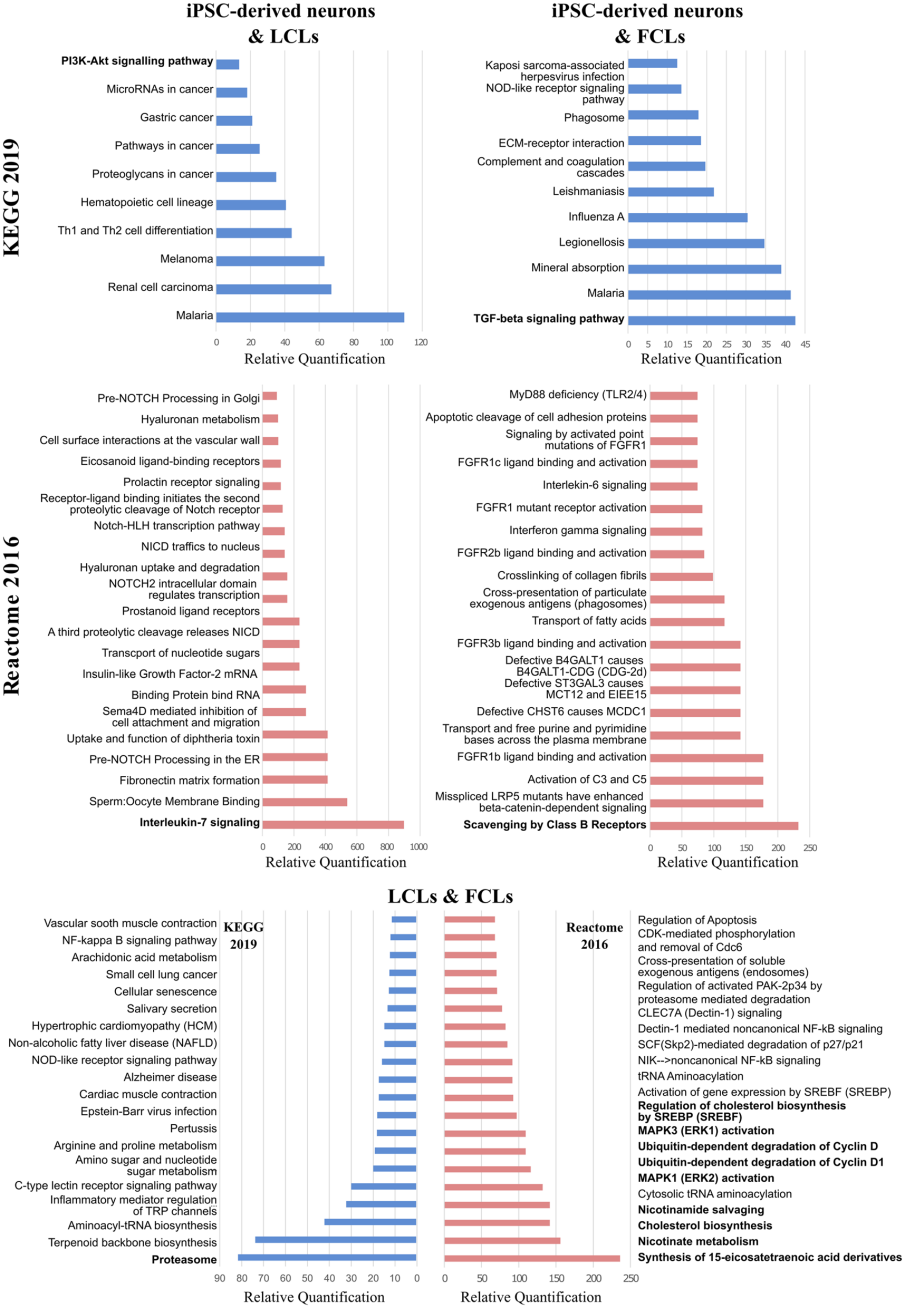
The top KEGG 2019 and Reactome 2016 pathways based on the combined score of EnrichR for the common DEGs (p-value < 0.05 and absolute log2FC > 1.2) of iPSC-derived neurons/LCLs, iPSC-derived neurons/FCLs and FCLs/LCLs respectively

**Fig. 5:**
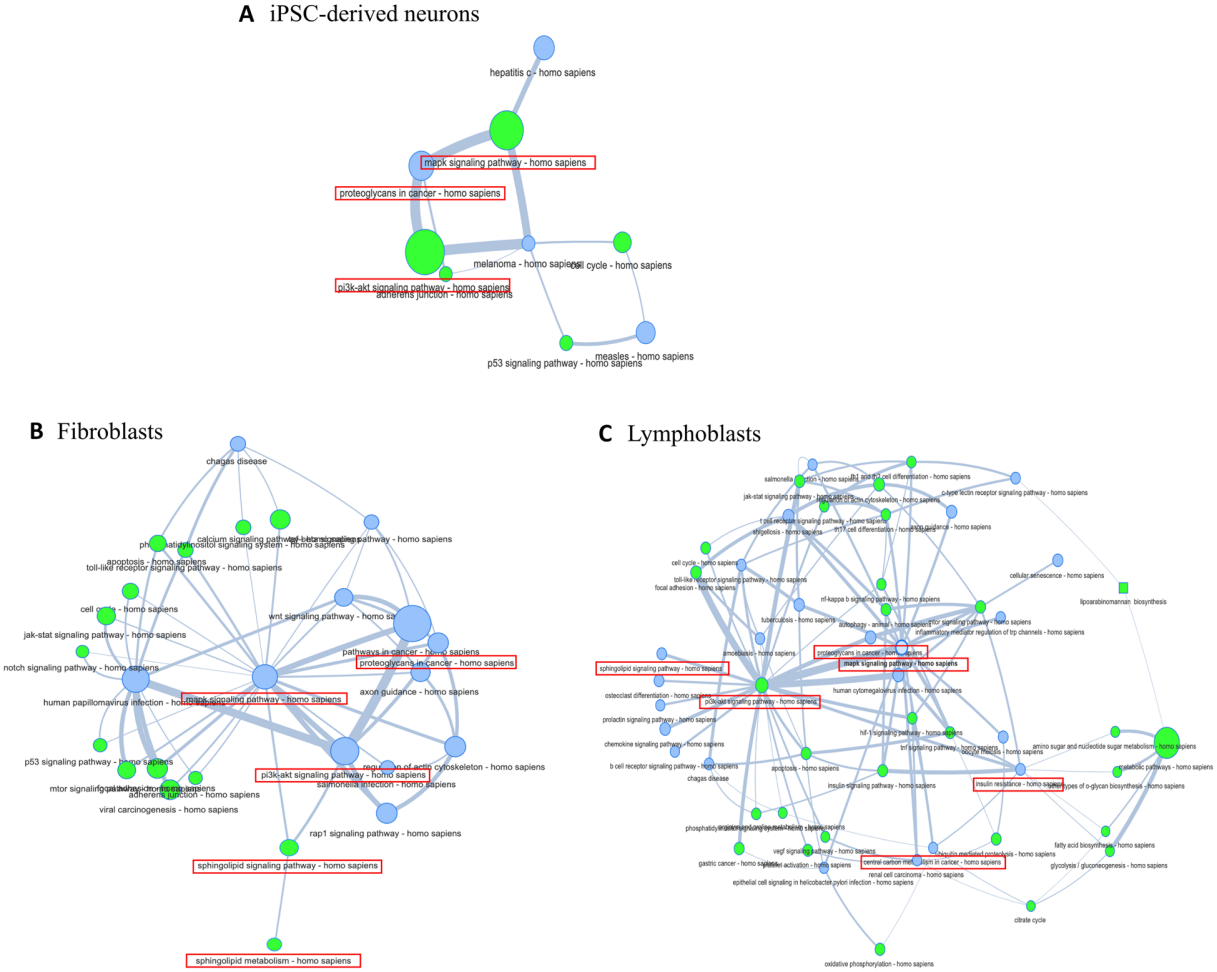
Pathway-to-pathway construction using the common pathways between the enrichment analysis of the top 500 DEGs of each tissue and the results of our previous study on gene expression datasets from ataxia and spasticity phenotypes. The common pathways between the two studies are presented in blue nodes. Additional pathways were included using a shortest path algorithm to either fill missing connections between pathway nodes or to enrich the pathway network. The most important pathways that could be associated with SA based on previous bibliography are designated in red

The “Proteoglycans in cancer”, “Pathways in cancer” and “PI3K-Akt signaling pathway” were found in the list of the top eight most enriched KEGG pathways resulting from the common DEGs (p-value < 0.05 and absolute log_2_FC > 1.2) of iPSC-derived neurons and LCLs. In addition, “Interleukin-7 signaling” was found as the most enriched Reactome 2016 pathway. For the iPSC-derived neurons and FCLs pair, the most enriched pathway based on KEGG 2019, was the “TGF-beta signaling pathway” (Fig. 4), while for Reactome 2016, it was the “Scavenging by class B receptors”. Analysis using the common DEGs of the pair FCLs and LCLs, for which there was a larger sample size (two patients vs two controls and three patients vs three controls, respectively) resulted in more pathways (Additional file 9). The top scored KEGG 2019 pathway was “Proteasome” while for Reactome 2016 was “Synthesis of 15-eicosatetraenoic acid derivatives”. Additional interesting pathways possibly relevant with the disease are indicated in Fig. 5 which summarizes all identified pathways. The full lists of the identified pathways are presented in Additional file 9.

### Discovery of common pathways between GBA2-associated SA and other ataxia/ spasticity phenotypes

As the name of the disease denotes, SA is mainly characterised by ataxia and spasticity features. Therefore, our current data could be compared with data resulting from other ataxia and/or spasticity phenotypes studies, in order to investigate whether there are shared pathways. For this reason, the current study KEGG 2019 resulting pathways of each tissue were compared to the identified pathways from our previous work [10], which was based on the analysis of multiple gene expression microarray datasets from ataxia and spasticity phenotypes. More specifically, gene expression microarray datasets from neuronal, peripheral blood and FCLs of patients with ataxia or spasticity phenotypes and control individuals were analysed and used to identify SA-associated pathways through our previous work [10]. Pathway comparison between the two studies revealed: (a) Four common pathways between the current iPSC-derived neurons and the previous neuronal tissue datasets analyses (the “MAPK signaling pathway”, “Melanoma”, “Measles” and “Proteoglycans in cancer”), (b) Twelve common pathways between the current and previous FCLs dataset analyses, (including the “TGF-beta”, “MAPK”, “Rap1” and “PI3K-Akt signaling” pathways, which are all connected to each other and to Sphingolipid pathways according to KEGG, Fig. 5), and (c) A total of 22 common pathways between the current LCLs and the previous peripheral blood datasets analyses. These include the “Sphingolipid signaling pathway”, in which GBA2 participates, “Axon guidance” that is associated with neuronal function, as well as the “Insulin resistance” and “Proteoglycans in cancer”. The “Insulin resistance” pathway could be of interest, since it was previously described that a cross-talk between sphingolipids and the insulin-like growth factor (IGF-I) might play a role in the control of cell survival, response to stress and aging, all of which are implicated in neurodegeneration [62].

Pathway connector was then used to generate pathway-to-pathway networks using the common KEGG 2019 pathways as input. Complementary pathways were obtained using a shortest path algorithm in order to fill any missing connection between our pathway nodes or even to enrich the already connected networks. The complementary pathways are shown in Fig. 5 as nodes in green colour. The size of the node represents the number of genes participating in the pathway, while the thickness of connections (edges) is directly proportional to the number of shared genes between the pathway pairs. The four initial pathways resulting from the comparison of the current iPSC-derived neurons with previous work corresponding tissue are shown in blue colour nodes (Fig. 5). The missing connections between these pathways have been filled with the addition of “MAPK signaling pathway”, “PI3K-Akt signaling pathway”, “Adherens junction”, “Cell cycle” and “p53 signaling pathway” (green colour nodes), as the pathways with the closest association based on a shortest-path algorithm. Regarding FCLs, among the supplementary pathways added in order to discover more associations that could be interesting in relation to SA have been the “Sphingolipid signaling” and “Sphingolipid metabolism” as well as the “Apoptosis” and the “Calcium signaling” pathways. Sphingolipid relevant pathways are directly correlated with *GBA2*-associated SA, due to the catalytic role of GBA2 in the sphingolipid metabolism, while apoptosis and calcium signaling have been associated with several neurodegenerative diseases based on bibliography [63–66]. Finally, in the LCLs network, among the additional pathways the “PI3K-Akt signaling pathway” (that is connected to several other neighbouring pathways, including the “Sphingolipid signaling pathway”), the “Autophagy”, the “MAPK signaling pathway” the “Proteoglycans in cancer” have been revealed.

## Discussion

Spastic ataxias (SAs) encompass a group of rare and severe neurodegenerative diseases, mainly characterized by limbs spasticity, imbalance and incoordination in gait and speech. Variants in the *GBA2* gene have been associated with SA, autosomal recessive spastic paraplegia type 46 (SPG46) and Marinesco-Sjögren syndrome (MSS). However, the pathogenetic mechanisms by which the specific *GBA2* variants lead to the development of the disease remain unclear. A few studies have examined the functional role of *GBA2* and reported its involvement in the sphingolipid metabolism and the hydrolysis of bile acid–3-O-β-glucosides (BG) [67]. In addition, it was shown through in-vitro studies that several *GBA2* variants, including the c.1780G > C missense variant, caused reduction of enzymatic activity in COS7 and HeLa cells [8]. Reduced activity of GBA2 was further confirmed in LCLs of SA patients, homozygous for the *GBA2* c.1780G > C missense variant [7]. Furthermore, it has been shown that C-terminally truncated variants move to mitochondria and cause mitochondrial fragmentation and loss of transmembrane potential [9]. The association of glucosylceramidases with neurodegeneration has also been recently described in relation to PD. Specifically, it has been shown that the activity of GBA and GBA2 progressively decrease in the substantia nigra with normal ageing and are further decreased in Parkinson’s disease (PD), followed by accumulation of glucosylceramide, which is the natural substrate of glucosylceramidases [68]. Another study has demonstrated that inhibition of GBA2 function in isolated cerebellar neurons dramatically affected F-actin dynamics and reduced neurite outgrowth [69].

Less information exists in relation to the overall SA pathogenetic mechanisms. Previous studies examined the gene expression at the RNA level in human tissues of ataxia patients and different ataxia mouse models, concluding to several candidate pathways, such as the glutamate signaling, the calcium signaling, the DNA repair, the cell cycle and various metabolic processes [70–76]. Moreover, transcriptome analysis on sacsin knockout mice showed disrupted expression of genes that participate in the processes of RNA processing, cell cycle, oxidative phosphorylation, autophagy and other [77]. However, to our knowledge, none of these studies aimed to identify implicated pathways specifically for SA in human, nor has been focused on *GBA2*-associated SA. Due to the sparse availability of information on the mechanisms by which variant *GBA2* leads to the development of the disease, more studies are needed to understand the functional role of *GBA2*. For this reason, we have performed the first whole coding genome transcriptomics analysis on iPSC-derived neurons, FCLs and LCLs from SA patients homozygous for the *GBA2* c.1780G > C missense variant and control individuals, and we have recorded the most significant pathways resulting from enrichment analysis of the identified DEGs.

Differential expression analysis revealed a total of 5217 genes with significantly altered expression in SA patient cells (iPSC-derived neurons, FCLs and LCLs) compared to controls. Among the DEGs there are known HCA and HSP genes, including the *SLC1A3* and *STUB1* which are more likely to be involved in common pathway(s) with *GBA2* according to their reported function. *SLC1A3* that was found over-expressed in both iPSC-derived neurons and FCLs, codes for a member of the high affinity glutamate transporter family, which are proteins involved in the neurotransmission in the CNS. Among its associated pathways are the neurotransmitter release cycle, synaptic vesicle cycle, transport of glucose and other sugars, bile salts and organic acids, metal ions and amine compounds, which are associated with neuronal function as well as glucose and lipid metabolism. The *STUB1* gene was found deregulated in FCLs and LCLs. *STUB1* codes for an E3 ubiquitin-protein ligase that participates in the TGF-β receptor signaling, which was found enriched through the current pathway analysis. Furthermore, comparison of the DEGs across the three different tissues revealed five common protein coding genes which in their majority are reported to be associated with nervous system diseases or pathways possibly involved in neuronal function. Among these genes the *CYB5R2* and *GLDC* are more likely to be involved in common pathway(s) with *GBA2*. Interestingly, CYB5R2 is a key player in lipid metabolism, catalysing the desaturation and elongation of fatty acids and cholesterol biosynthesis. The sphingolipid metabolism, in which GBA2 participates, is part of the lipid metabolism. Furthermore, GLDC participates in the metabolism of glycine, serine and threonine, which belong in the generic pathway of “Metabolism of amino acids and derivatives” according to Reactome. Its role to catalyse the degradation of glycine can affect the fatty acid response and therefore it is also associated with the metabolism of lipids.

Two pathway analysis approaches were performed for the discovery of SA pathways using the lists of identified DEGs in patient samples compared to controls. The first approach was focused on the analysis of the top 500 DEGs (over- and under-expressed) per tissue (iPSC-derived neurons, FCLs, LCLs), followed by comparisons of the identified pathways. These results indicated a minimal number of common pathways between the three tissues, suggesting the possibility that tissue specific pathological mechanisms might exist or some genes have tissue specific expression. Nevertheless, these could be indeed the most significant pathways, or limitations of the current study concerning the sample size could have an effect on the results. Interestingly, differential pathway identification not only between tissues but also between different brain regions, has been reported through other neurodegenerative disease expression studies [78, 79], thus partly supporting our suggestion.

The currently identified pathways that are common between the three studied tissues are the “Proteoglycans in cancer” (KEGG 2019) and “Cytokine Signaling in Immune system” (Reactome 2016). Cancer and neurodegenerative diseases share several common biological mechanisms and pathways, such as dysregulation in metabolism, oxidative stress and mitochondrial dysfunction, as well as inflammation, leading to an apoptotic effect in neurodegeneration and an anti-apoptotic effect in cancer [80]. The KEGG pathway “Proteoglycans in cancer” consists a sum of several pathways including the “MAPK signaling pathway”, the “TGF-β signaling pathway”, the “PI3K-Akt signaling pathway”, the “Calcium signaling pathway”, the “Wnt signaling pathway” and “Apoptosis” [81]. All of these pathways share common players, which were found differentially expressed in our transcriptomic analysis, such as the *NGFR*, *CCND2*, *FGF2* and *MAPKAPK3* genes (Additional file 8). On the other hand, changes in cytokine signaling have been suggested as a common mechanism in the development and pathogenesis of several neurodegenerative diseases. The TGF-β is an example of a cytokine that is known to be associated with neurodegeneration in AD [82]. Moreover, the “TGF-β signaling pathway” has been found as one of the most enriched pathways through some of the approaches performed.

In addition to the above, under the hypothesis that the iPSC-derived neurons are the closest to the disease primarily affected tissues, emphasis on this tissue findings and the relevant first approach pairwise comparisons derived pathways, was given. Moreover, our second approach, aiming towards the identification of more possible interesting pathways, was based on the use of the common DEGs between the tissues in pairs as inputs. Several candidate pathways resulted from the pairwise comparisons of both approaches, especially from the second, relevant with inflammation, sialylation, glucosylation, glycosaminoglycan metabolism, lipid metabolism and signal transduction. We hereby discuss the top scored pathways identified through the neurons analysis and some of the most significant, selected through the other comparison findings. Our selection is based on a combination of the resulting enrichment score and the existing knowledge supporting a possible relation with the nervous system function. Therefore, through the neurons analysis (Additional file 8), “interferon signaling” and “RIG-I-like receptor signaling” have been the top-scored Reactome 2016 and KEGG 2019 pathways respectively, suggesting that neuroinflammation might play an important role in the pathogenesis of SA similarly to other neurodegenerative diseases, such as AD [83], FRDA [84, 85] and ALS [86, 87] or it may even be the reaction of the cells to the altered function of GBA2. In addition, RIG-I signaling has been found significantly increased in the plasma and temporal cortex of mild cognitive impairment patients with AD, as well as in the occipital cortex of AD patients. Elevation of RIG-I can be caused by small self-RNA cleavage products or by ROS, which are in turn associated with oxidative stress [83].

Comparison of the iPSC-derived neurons and LCLs resulting pathways through the first approach, also provided immune system and interferon alpha/beta signaling (Table 3) as their only common pathways, thus further supporting the involvement of inflammation in *GBA2*-related neurodegeneration. On the other hand, one of the most interesting common pathways identified through the pathway comparison of iPSC-derived neurons and FCLs has been the “glycosaminoglycan (GAG) metabolism” thus supporting a possible involvement of the GAGs in the disease. Disturbances in the metabolism of GAGs have been previously characterised in lysosomal storage disorders [88]. GAGs can lead to the generation of protein aggregates and in turn accumulation in several tissues including the brain. Furthermore, they have a role in inflammation and have been reported in a number of neurodegenerative disorders, such as PD and AD [89].

Enrichment analysis of the iPSC-derived neurons and FCLs common DEGs, identified the “Scavenging by class B receptors”, as the most enriched Reactome 2016 pathway and the “TGF-β signaling pathway” as the most enriched according to KEGG 2019. Scavenger receptors describe a type of pattern recognition receptors found on macrophage surface that mediate endocytosis of acetylated low-density lipoproteins (LDL). Class B scavenger receptors have been previously described to play a role in AD. Specifically, the CD36 class B scavenger receptor binds to Αβ and stimulates the production of cytokines and chemokines from microglia, as well as ROS through Src signaling and MAPK activation [90]. In addition, the scavenger receptor class B type I (SR-BI), that is expressed in astrocytes and smooth muscle cells of human adult brain, is known for its role in cholesterol turnover and is also suggested to participate in the pathogenesis of AD [91]. Disturbances in TGF-β signaling have been previously shown to promote neurodegeneration and AD pathology [92]. Specifically, decreased levels of neuronal TGF-β signaling was shown to increase Ab levels, amyloid deposition and dendritic degeneration in mice expressing the human APP protein [92]. In addition, aging and chronic inflammation were shown to reduce the canonical TGF-β1/SMAD signaling, facilitating cytotoxic activation of microglia and microglia-mediated neurodegeneration [93].

Analysis of the iPSC-derived neurons and LCLs common DEGs revealed the “PI3K-Akt signaling pathway” (KEGG 2019) among others (including “Proteoglycans in cancer”), which could possibly be the most relevant to the nervous system function even though not being the top scored. The “PI3K-Akt signaling pathway” plays a role in various biological processes, such as metabolism, proliferation, cell survival, growth and angiogenesis [94]. Disturbances in PI3K-Akt/mTOR signaling in neurons lead to increased ROS levels, membrane depolarization, mitochondrial fragmentation, decreased oxidative phosphorylation, and lower ATP production [95–97]. Furthermore, gene expression studies in spinocerebellar ataxia 3 (SCA3) mouse models revealed that the PI3K-Akt signaling pathway was enriched based on genes with altered expression in SCA3 mice brainstem [98]. According to Reactome results, “Interleukin-7 signaling” has been identified as the most enriched pathway. Interleukin-7 (IL-7) is a cytokine that regulates the survival, proliferation and differentiation of lymphocytes [99]. Its role is essential for the development of both adaptive and innate immunity lymphocytes [100], while previous reports have associated IL-7 signaling with multiple sclerosis through its effect in the dysregulation of N-glycosylation [101, 102]. Moreover, transcriptional analysis of FRDA patient peripheral blood samples showed deregulation of inflammatory genes linked to innate immunity [84]. It has been also reported that IL-7 stimulates PI3K-Akt signaling to phosphorylate and inactivate the pro-apoptotic protein Bad, thus promoting cell survival [103]. Moreover, as already discussed above, apart from the immune response, neuroinflammation has been associated with neurodegeneration. Therefore, based on our findings we can suggest their possible involvement in the *GBA2*-associated SA pathogenesis. However, it remains to be determined whether they contribute to the disease pathology, or whether they are just subsequent responses to stresses induced by other dysregulating mechanisms.

Our initial focus was on the results that involved the iPSC-derived neurons derived data, as they are the closest to the primarily affected tissues in the studied disease. However, since the limited number of samples [1 patient vs 1 control] could possibly affect the identification of significant findings and under the hypothesis that tissue specific pathological mechanisms might exist, a pairwise analysis of the FCLs and LCLs was also considered as significant. Indeed, this analysis enabled the identification of additional interesting findings that could be associated with neurodegeneration, based on the existing knowledge. These include the top scored KEGG 2019 pathway “Proteasome”, the top scored Reactome 2016 pathway “Synthesis of 15-eicosatetraenoic acid derivatives”, as well as the “Nicotinate metabolism”, “Cholesterol biosynthesis”, “Nicotinamide salvaging”, “MAPK1/3 (ERK2/1) activation”, “Ubiquitin-dependent degradation of Cyclin D/D1” and “Regulation of cholesterol biosynthesis by SREBP”, which interestingly have been among the top ten enriched Reactome 2016 pathways, and some of them can be grouped under more common general pathways as discussed below.

The ubiquitin–proteasome system (UPS), supported by both databases findings, is responsible for protein degradation. Its dysregulation can result to neurotoxic protein accumulation and aggregation, a major characteristic of several neurodegenerative diseases, such as AD, PD, and Huntington's disease (HD) [104]. The “Synthesis of 15-eicosatetraenoic acid derivatives” consists part of the “arachidonic acid metabolism”. The 15-eicosatetraenoic acids are formed after oxidation of arachidonic acid, a polyunsaturated fatty acid (PUFA) that is abundant in the brain. Arachidonic acid has been previously reported to be involved through several mechanisms (including initiation and progression of neuroinflammation) in AD [105], and to have a protective role in PD in vitro models [106]. Decreased levels of this and other PUFAs have also been associated with spinocerebellar ataxia 38 pathogenesis [107, 108]. Furthermore, it has been associated with oxidative stress and lipid peroxidation [109, 110]. Therefore, the current “arachidonic acid metabolism” indirect identification, further supports that lipid metabolism might play a role in SA pathogenesis and that previously reported neurodegeneration mechanisms, such as oxidative stress, lipid peroxidation and neuroinflammation might also be involved.

The “Nicotinate and nicotinamide metabolism” describes a general pathway of vitamins and cofactors metabolism and involves the nicotinamide-adenine dinucleotide (NAD+) and nicotinamide-adenine dinucleotide phosphate (NADP+) precursors, nicotinate and nicotinamide (also known as vitamin B3). The NAD+ and NADP+ are essential coenzymes in redox reactions of respiration and are thus involved in energy production, while they also participate in other biological processes, such as gene expression, cell cycle, DNA repair and cell death [111]. Both niacin and its derivative nicotinamide have a neuroprotective role and they have been implicated in neurodegenerative diseases, such as AD, PD and HD [111, 112]. Furthermore, nicotinate and nicotinamide, among other vitamins and cofactors have been described for their potential contribution in the treatment of oxidative stress-induced neuromuscular diseases [113].

“Cholesterol biosynthesis” is part of the lipid metabolism; cholesterol, together with sphingolipids and phospholipids are key elements of membrane lipid bilayers [114]. Under the hypothesis that the sphingolipid metabolism could be affected in SA, the regulation of the general lipid metabolism might be affected as well. The production of cholesterol is vital for the cell, as it is required for the biogenesis and maintenance of the plasma membrane, as well as the transmembrane communication between cellular compartments [115]. In the brain, cholesterol acts as a precursor molecule for the production of steroid hormones and myelin. Many neurodegenerative diseases, such as AD, PD, ALS, SCA3 and Niemann-Pick disease type C are characterized by impaired cholesterol turnover in the brain [115–119]. The relation of cholesterol to mitochondria has also been under the spotlight regarding neurodegeneration. Mitochondria require cholesterol for membrane synthesis as well, but also for the production of steroids, oxysterols and hepatic bile acids. In vitro studies in model systems of cholesterol-enriched mitochondria and mitochondria isolated from cells with pathophysiological cholesterol accumulation, were used to show that disturbances in the amount of mitochondrial cholesterol affect the mitochondrial function. Elevated levels of cholesterol have been observed in several neurodegenerative diseases [120].

Finally, the “MAPK signaling pathway” has been previously associated with a number of neurodegenerative diseases, including AD, PD and ALS, while it also contributes to neuroinflammation. Studies have also shown that the molecular mechanisms behind neuronal damage induced by oxidative stress involve a death mode in which MAPK signaling pathways are strongly implicated [121, 122].

In addition to the above pathway analyses, the currently produced data have been further compared with our previous work data [10] towards the possible identification of more pathways and/or the strengthening of the current findings. The most significant findings of our previous work included the Sphingolipid metabolism and signaling pathway, the PI3K-Akt signaling pathway, the MAPK signaling pathway, the Calcium signaling and mitochondrial-associated pathways, as well as the Insulin signaling pathway. Comparison of these data with the current, enabled the extraction of common findings. Interestingly, “Proteoglycans in cancer” consistently appeared through pathway comparisons of all the current tissues (Fig. 5) with the relevant previous work tissues, while the “MAPK signaling” was identified through FCLs and LCLs comparisons. The “Sphingolipid signaling pathway” was also identified through LCLs comparisons, while the “PI3K-Akt signaling” resulted from the FCLs comparisons. Subsequent pathway to pathway networks constructions for all the tissues and addition of supplementary pathways in order to fill in any missing connections between the pathway nodes and to discover additional pathway connections added more findings: the “MAPK signaling pathway” in iPSC-derived neurons, the “Sphingolipid signaling pathway” and “Sphingolipid metabolism” in FCLs, and the “PI3K-Akt signaling pathway” as a complementary in FCLs and LCLs (Fig. 5). Overall, the “MAPK signaling pathway” that was identified in all three tissues analysed, shows a direct connection with “PI3K-Akt” and “mTOR signaling” pathways, while these pathways also share an association with “Sphingolipid metabolism” and “apoptosis”. Overall, the comparison of the results of the current study and our previous work has strengthen the possible involvement of the above-mentioned pathways in the pathogenesis of *GBA2*-caused SA and highlighted possible pathway synergies that could be further examined to draw valid conclusions.

Due to the rarity of *GBA2*-caused SA, this work presents information based on a limited sample size of iPSC-derived neurons (one patient and one control), FCLs (two patients and two controls) and LCLs (three patients and six controls). For this reason, two different approaches of pathway analysis were performed, and the results were evaluated and compared with our previous work on a large number of ataxia and spasticity phenotype gene expression datasets, as well as the bibliography. This work discusses the possible implication of the identified pathways in *GBA2*-caused SA pathogenesis; nevertheless, additional studies should be performed using a larger sample of patients, in order to establish stronger connections of the suggested pathways to the disease.

## Conclusions

In conclusion, our work examines for the first time the transcriptome profile of SA patients homozygous for the *GBA2* c.1780G > C variant, as well as of control individuals, and presents the identification of DEGs through patient-control comparisons. In addition, pathway analyses using the DEGs data highlight the most interesting pathways as candidates for the *GBA2*-associated SA, after performing further assessment using our previous work data and bibliography reports. Our results support the implication of oxidative stress to the disease that has previously been proposed as a pathogenic mechanism in other neurodegenerative diseases, and also provide information about the possible involvement of the MAPK, the PI3K-Akt/mTOR signaling pathways, as well as the neuroinflammation and the lipid metabolism dysregulation. In addition, our work strengthens the implication of the sphingolipid metabolism, in which *GBA2* participates, and supports a possible cross-talk between the sphingolipid metabolism with some of the proposed pathways, as indicated by the in-silico network analysis. These results suggest that several pathways rather than a single, could be deregulated thus leading to the SA phenotype. Since some of the pathways were found to be connected through the in-silico approach performed, the effect of each pathway deregulation, as well as the synergies between these pathways could be investigated and confirmed or excluded through experimental studies. Moreover, because of the limitation of the small sample size that is included in the current study, additional similar or even larger sample-sized gene expression studies on SA patients diagnosed with *GBA2* mutations could be performed, thus enabling comparison with the current findings or repetition of the data analyses after pooling more sample data (including this study samples). Furthermore, in case of lack of additional SA patient data, animal models and/or human cell lines could be used to functionally study the proposed pathways in disease and compare with control state. Such approaches might enable the extraction of more specific and significant findings including a possible exclusion of some pathways or the identification of novel. Furthermore, this study provides a list of common DEGs and pathways within tissues of SA patients that could be further validated in a larger sample size and in additional more easily accessible tissues (i.e. blood) towards the discovery of disease biomarkers. Overall, our work indicates *GBA2*-associated SA candidate pathways in human for the first time, and provides knowledge that could contribute to the disease pathogenetic mechanism determination.

## Supplementary Information

**Additional file 1: Figure S1.** A) Bright-field microscopy of CTRL DANs at 45 days of differentiation showing typical morphology with elongated and branching processes B) ICC of the pan-neuronal marker TUJ1 expressed by CTRL DANs at 35 days of differentiation C) ICC showing expression of the neuronal (TUJ1) and dopaminergic (TH) markers in DANs cultures at 50 days of differentiation from one control and one patient’s line. Most neuronal cells exhibit co-expression of TUJ1 and TH, demonstrating their dopaminergic identity. Abbreviations: CTRL: control, DANs: dopaminergic neurons, TH: tyrosine hydroxylase, TUBB3: beta-III tubulin.

**Additional file 2. ** Mapping percentages. The file includes the percentage of RNA-Seq reads mapped successfully against the reference genome for each sample analyzed.

**Additional file 3. **Raw counts. The file contains the raw counts produced for each gene mapped against the reference genome.

**Additional file 4. **Differentially expressed genes. The file contains the list of differentially expressed genes from the comparison between patients and control individuals for each cell line (LCLs, FCLs, iPSC-derived neurons). The gene name is provided, along with the fold change (FC), the logFC, the log counts per million (CPM), the F-value provided by the statistical test, the P-value, the false discovery rate (FDR) and the absolute logFC (abslogFC).

**Additional file 5. **Validation of selected genes—real-time PCR. The file contains the fold change (FC) and p-value of selected DEGs calculated from RNA-Seq and real-time PCR experiments. Statistical analysis was performed only for the LCL tissue.

**Additional file 6. **HCA and HSP genes. The file contains HCA and HSP genes found differentially expressed in each of the three cell lines analyzed (LCLs, FCLs, iPSC-derived neurons).

**Additional file 7. **Common DEGs between the three cell lines. The file contains the common DEGs between the three cell lines and also between each pair of cell lines.

**Additional file 8. **Enrichment analysis of the top 500 DEGs. The file contains the pathways that resulted from the enrichment analyses of the top 500 DEGs of each of the three cell lines analyzed (LCLs, FCLs, iPSC-derived neurons).

**Additional file 9. ** Enrichment analysis of the common DEGs between cell lines. The file contains the pathways that resulted from the enrichment analyses of the common DEGs between each pair of cell lines.

## Data Availability

All data generated or analysed during this study are included in this published article and its supplementary files.

## Abbreviations

AD: Alzheimer disease
ALS: Amyotrophic lateral sclerosis
Aβ: Beta amyloid
BDNF: Brain-derived neurotrophic factor
BG: Bile acid-3-O-β-glucosides
CF: Control fibroblast
CGN: Cultured cerebellar granule neurons
CL: Control lymphoblast
CMT: Charcot–Marie–Tooth
CPM: Count per million
CTRL: Control
DANs: Dopaminergic neurons
DE: Differential expression
DEG: Differentially expressed gene
DMD: Duchenne muscular dystrophy
EBV: Epstein–Barr virus
FCL: Fibroblast cell line
FDR: False-discovery rate
FRDA: Friedreich’s ataxia
GAG: Glycosaminoglycan
GBA: Lysosomal glucosylceramidase
GBA2: Non-lysosomal glucosylceramidase
GDNF: Glial cell line-derived neurotrophic factor
GlcCer: Glucosylceramide
HCA: Hereditary cerebellar ataxia
HD: Huntington's disease
HSP: Hereditary spastic paraplegia
IGF-I: Insulin-like growth factor
IL-7: Interleukin-7
iPSC: Induced pluripotent stem cell
LCL: Lymphoblastoid cell line
MSS: Marinesco–Sjögren syndrome
NAD+: Nicotinamide-adenine dinucleotide
NADP+: Nicotinamide-adenine dinucleotide phosphate
PD: Parkinson disease
PF: Patient fibroblast
PL: Patient lymphoblast
PUFA: Polyunsaturated fatty acid
QC: Quality control
RNA-Seq: RNA-sequencing
SA: Spastic ataxias
SCA3: Spinocerebellar ataxia 3
SPG46: Spastic paraplegia type 46
SR-BI: Scavenger receptor class B type I
TH: Tyrosine hydroxylase
TMM: Trimmed mean of M-values
TUBB3: Beta-III tubulin
UPS: Ubiquitin–proteasome system
WT: Wild type

## References

[1] Parodi L, Coarelli G, Stevanin G, Brice A, Durr A (2018). Hereditary ataxias and paraparesias: clinical and genetic update. Curr Opin Neurol..

[2] De Bot ST, Willemsen MAAP, Vermeer S, Kremer HPH, VanDeWarrenburg BPC (2012). Reviewing the genetic causes of spastic-ataxias. Neurology.

[3] Minnerop M, Kurzwelly D, Wagner H, Soehn AS, Reichbauer J, Tao F (2017). Hypomorphic mutations in POLR3A are a frequent cause of sporadic and recessive spastic ataxia. Brain.

[4] Calandra CR, Buda G, Vishnopolska SA, Oliveri J, Olivieri FA, Pérez Millán MI, et al. Spastic ataxia with eye-of-the-tiger-like sign in 4 siblings due to novel compound heterozygous AFG3L2 mutation. Parkinson Relat Disord. 2020; 73:52–4. http://www.prd-journal.com/article/S1353802020300766/fulltext. Accessed 14 Feb 2021. 10.1016/j.parkreldis.2020.03.020 32248051

[5] Votsi C, Zamba-Papanicolaou E, Middleton LT, Pantzaris M, Christodoulou K (2014). A novel GBA2 gene missense mutation in spastic ataxia. Ann Hum Genet.

[6] Harzer K, Blech-Hermoni Y, Goldin E, Felderhoff-Mueser U, Igney C, Sidransky E (2012). Beta-glucosidase 1 (GBA1) is a second bile acid β-glucosidase in addition to β-glucosidase 2 (GBA2) Study in β-glucosidase deficient mice and humans. Biochem Biophys Res Commun..

[7] Malekkou A, Samarani M, Drousiotou A, Votsi C, Aureli M, Loberto N (2018). Biochemical characterization of the GBA2 c. 1780G > C missense mutation in lymphoblastoid cells from patients with spastic ataxia. IJMS..

[8] Sultana S, Reichbauer J, Schüle R, Mochel F, Synofzik M, Van Der Spoel AC (2015). Lack of enzyme activity in GBA2 mutants associated with hereditary spastic paraplegia/cerebellar ataxia (SPG46). Biochem Biophys Res Commun.

[9] Sultana S, Stewart J, van der Spoel AC (2020). Truncated mutants of beta-glucosidase 2 (GBA2) are localized in the mitochondrial matrix and cause mitochondrial fragmentation. PLoS ONE.

[10] Kakouri AC, Votsi C, Tomazou M, Minadakis G, Karatzas E, Christodoulou K (2020). Analyzing gene expression profiles from ataxia and spasticity phenotypes to reveal spastic ataxia related pathways. Int J Mol Sci.

[11] MonzioCompagnoni G, Kleiner G, Samarani M, Aureli M, Faustini G, Bellucci A (2018). Mitochondrial dysregulation and impaired autophagy in iPSC-derived dopaminergic neurons of multiple system atrophy. Stem Cell Rep..

[12] Kriks S, Shim JW, Piao J, Ganat YM, Wakeman DR, Xie Z (2011). Dopamine neurons derived from human ES cells efficiently engraft in animal models of Parkinson’s disease. Nature.

[13] Andrews S. FASTQC A quality control tool for high throughput sequence data. Babraham Inst; 2015. http://www.bioinformatics.babraham.ac.uk/projects/fastqc/.

[14] FASTX-Toolkit FASTQ/A short-reads pre-processing tools. http://hannonlab.cshl.edu/fastx_toolkit/.

[15] Langmead B, Trapnell C, Pop M, Salzberg SL (2009). Ultrafast and memory-efficient alignment of short DNA sequences to the human genome. Genome Biol..

[16] Kent WJ, Sugnet CW, Furey TS, Roskin KM, Pringle TH, Zahler AM (2002). The human genome browser at UCSC. Genome Res.

[17] Langmead B, Trapnell C, Pop M, Salzberg SL (2009). Ultrafast and memory-efficient alignment of short DNA sequences to the human genome. Genome Biol.

[18] Anders S, Pyl PT, Huber W (2015). HTSeq—a Python framework to work with high-throughput sequencing data. Bioinformatics.

[19] Robinson MD, McCarthy DJ, Smyth GK (2009). edgeR: a bioconductor package for differential expression analysis of digital gene expression data. Bioinformatics.

[20] Lun ATL, Chen Y, Smyth GK (2016). It’s DE-licious: a recipe for differential expression analyses of RNA-seq experiments using quasi-likelihood methods in edgeR. Methods Mol Biol.

[21] Minadakis G, Zachariou M, Oulas A, Spyrou GM (2019). PathwayConnector: finding complementary pathways to enhance functional analysis. Bioinformatics.

[22] Depondt C, Donatello S, Rai M, Wang FC, Manto M, Simonis N (2016). MME mutation in dominant spinocerebellar ataxia with neuropathy (SCA43). Neurol Genet.

[23] Higuchi Y, Hashiguchi A, Yuan J, Yoshimura A, Mitsui J, Ishiura H (2016). Mutations in *MME* cause an autosomal-recessive Charcot–Marie–Tooth disease type 2. Ann Neurol.

[24] Hong D, Fang P, Yao S, Chen J, Zhang X, Chen S (2019). Variants in *MME* are associated with autosomal-recessive distal hereditary motor neuropathy. Ann Clin Transl Neurol.

[25] Desai S, Juncker M, Kim C (2018). Regulation of mitophagy by the ubiquitin pathway in neurodegenerative diseases. Exp Biol Med.

[26] Desai SD, Reed RE, Babu S, Lorio EA (2013). ISG15 deregulates autophagy in genotoxin-treated ataxia telangiectasia cells. J Biol Chem.

[27] Zafar F, Valappil RA, Kim S, Johansen KK, Chang ALS, Tetrud JW (2018). Genetic fine-mapping of the Iowan SNCA gene triplication in a patient with Parkinson’s disease. NPJ Park Dis..

[28] Xiu MX, Zeng B, Kuang BH (2020). Identification of hub genes, miRNAs and regulatory factors relevant for Duchenne muscular dystrophy by bioinformatics analysis. Int J Neurosci..

[29] Salvalaio M, D’Avanzo F, Rigon L, Zanetti A, D’Angelo M, Valle G (2017). Brain RNA-seq profiling of the mucopolysaccharidosis type II mouse model. Int J Mol Sci.

[30] Bai B, Wang X, Li Y, Chen PC, Yu K, Dey KK (2020). Deep multilayer brain proteomics identifies molecular networks in Alzheimer’s disease progression. Neuron.

[31] Iansante V, Choy PM, Fung SW, Liu Y, Chai JG, Dyson J (2015). PARP14 promotes the Warburg effect in hepatocellular carcinoma by inhibiting JNK1-dependent PKM2 phosphorylation and activation. Nat Commun.

[32] Bezprozvanny I, Mattson MP (2008). Neuronal calcium mishandling and the pathogenesis of Alzheimer’s disease. Trends Neurosci..

[33] Mastorci K, Montico B, Faè DA, Sigalotti L, Ponzoni M, Inghirami G (2016). Phospholipid scramblase 1 as a critical node at the crossroad between autophagy and apoptosis in mantle cell lymphoma. Oncotarget.

[34] Witt SH, Streit F, Jungkunz M, Frank J, Awasthi S, Reinbold CS (2017). Genome-wide association study of borderline personality disorder reveals genetic overlap with bipolar disorder, major depression and schizophrenia. Transl Psychiatry..

[35] Conde MA, Alza NP, Iglesias González PA, Scodelaro Bilbao PG, Sánchez Campos S, Uranga RM (2018). Phospholipase D1 downregulation by α-synuclein: Implications for neurodegeneration in Parkinson’s disease. Biochim Biophys Acta - Mol Cell Biol Lipids.

[36] Kon T, Miki Y, Tanji K, Mori F, Tomiyama M, Toyoshima Y (2015). Localization of nuclear receptor subfamily 4, group A, member 3 (NR4A3) in Lewy body disease and multiple system atrophy. Neuropathology.

[37] Goizet C, Boukhris A, Durr A, Beetz C, Truchetto J, Tesson C (2009). CYP7B1 mutations in pure and complex forms of hereditary spastic paraplegia type 5. Brain.

[38] Chen JY, Wu JF, Kimura A, Nittono H, Liou BY, Lee CS (2020). AKR1D1 and CYP7B1 mutations in patients with inborn errors of bile acid metabolism: possibly underdiagnosed diseases. Pediatr Neonatol..

[39] Goldsmith AP, Gossage SJ, Ffrench-Constant C (2004). ADAM23 is a cell-surface glycoprotein expressed by central nervous system neurons. J Neurosci Res..

[40] McGrath-Morrow SA, Ndeh R, Collaco JM, Rothblum-Oviatt C, Wright J, O’Reilly MA (2018). Inflammation and transcriptional responses of peripheral blood mononuclear cells in classic ataxia telangiectasia. PLoS ONE.

[41] Sakharkar MK, Singh SKK, Rajamanickam K, Essa MM, Yang J, Chidambaram SB (2019). A systems biology approach towards the identification of candidate therapeutic genes and potential biomarkers for Parkinson’s disease. PLoS ONE.

[42] Pflieger LT, Dansithong W, Paul S, Scoles DR, Figueroa KP, Meera P (2017). Gene co-expression network analysis for identifying modules and functionally enriched pathways in SCA2. Hum Mol Genet.

[43] Lan Y, Han J, Wang Y, Wang J, Yang G, Li K (2018). STK17B promotes carcinogenesis and metastasis via AKT/GSK-3β/Snail signaling in hepatocellular carcinoma. Cell Death Dis.

[44] Prasad GR, Jho E hoon. A concise review of human brain methylome during aging and neurodegenerative diseases. BMB Rep. 2019;52(10). 10.5483/BMBRep.2019.52.10.215 PMC6827576 31462381

[45] Ali F, Josephs KA (2018). Corticobasal degeneration: key emerging issues. J Neurol..

[46] Patron M, Sprenger HG, Langer T (2018). M-AAA proteases, mitochondrial calcium homeostasis and neurodegeneration. Cell Res.

[47] Martinelli P, Rugarli EI (2010). Emerging roles of mitochondrial proteases in neurodegeneration. Biochim Biophys Acta Bioenerg..

[48] You WD, Tang QL, Wang L, Lei J, Feng JF, Mao Q (2016). Alteration of microRNA expression in cerebrospinal fluid of unconscious patients after traumatic brain injury and a bioinformatic analysis of related single nucleotide polymorphisms. Chin J Traumatol..

[49] Kong W, Mou X, Liu Q, Chen Z, Vanderburg CR, Rogers JT (2009). Independent component analysis of Alzheimer’s DNA microarray gene expression data. Mol Neurodegener..

[50] Jiang D, Zhang Y, Hart RP, Chen J, Herrup K, Li J (2015). Alteration in 5-hydroxymethylcytosine-mediated epigenetic regulation leads to Purkinje cell vulnerability in ATM deficiency. Brain.

[51] Droppelmann CA, Campos-Melo D, Volkening K, Strong MJ, Volkening K, Strong MJ (2014). The emerging role of guanine nucleotide exchange factors in ALS and other neurodegenerative diseases. Front Cell Neurosci..

[52] Miyamoto Y, Yamauchi J (2010). Cellular signaling of Dock family proteins in neural function. Cell Signal.

[53] Detera-Wadleigh SD, Liu CY, Maheshwari M, Cardona I, Corona W, Akula N (2007). Sequence variation in DOCK9 and heterogeneity in bipolar disorder. Psychiatr Genet..

[54] Sanchez-Pulido L, Ponting CP (2018). TMEM132: An ancient architecture of cohesin and immunoglobulin domains define a new family of neural adhesion molecules. Bioinformatics.

[55] Davis CA, Dhawan IK, Johnson MK, Barber MJ (2002). Heterologous expression of an endogenous rat cytochrome b5/cytochrome b5 reductase fusion protein: identification of histidines 62 and 85 as the heme axial ligands. Arch Biochem Biophys.

[56] Bewley MC, Marohnic CC, Barber MJ (2001). The structure and biochemistry of NADH-dependent cytochrome b5 reductase are now consistent. Biochemistry.

[57] Hyun DH, Lee GH (2015). Cytochrome b5 reductase, a plasma membrane redox enzyme, protects neuronal cells against metabolic and oxidative stress through maintaining redox state and bioenergetics. Age (Omaha)..

[58] Samhan-Arias AK, Fortalezas S, Cordas CM, Moura I, Moura JJG, Gutierrez-Merino C (2018). Cytochrome b5 reductase is the component from neuronal synaptic plasma membrane vesicles that generates superoxide anion upon stimulation by cytochrome c. Redox Biol.

[59] Valencia A, Morán J (2004). Reactive oxygen species induce different cell death mechanisms in cultured neurons. Free Radic Biol Med..

[60] Yokoyama JS, Karch CM, Fan CC, Bonham LW, Kouri N, Ross OA (2017). Shared genetic risk between corticobasal degeneration, progressive supranuclear palsy, and frontotemporal dementia. Acta Neuropathol..

[61] Foguem C, Kamsu-Foguem B (2016). Neurodegeneration in tauopathies and synucleinopathies. Rev Neurol (Paris)..

[62] Jęśko H, Stępień A, Lukiw WJ, Strosznajder RP (2019). The cross-talk between sphingolipids and insulin-like growth factor signaling: significance for aging and neurodegeneration. Mol Neurobiol..

[63] Patten DA, Germain M, Kelly MA, Slack RS (2010). Reactive oxygen species: stuck in the middle of neurodegeneration. J Alzheimer’s Dis..

[64] Bezprozvanny IB (2010). Calcium signaling and neurodegeneration. Acta Nat.

[65] Alzheimer’s Association Calcium Hypothesis Workgroup (2017). Calcium Hypothesis of Alzheimer’s disease and brain aging: a framework for integrating new evidence into a comprehensive theory of pathogenesis. Alzheimer’s Dement.

[66] Vig PJ, Subramony SH, McDaniel DO (2018). Calcium homeostasis and spinocerebellar ataxia-1 (SCA-1). Brain Res Bull.

[67] Boot RG, Verhoek M, Donker-Koopman W, Strijland A, Van Marle J, Overkleeft HS (2007). Identification of the non-lysosomal glucosylceramidase as β-glucosidase 2. J Biol Chem.

[68] Huebecker M, Moloney EB, Van Der Spoel AC, Priestman DA, Isacson O, Hallett PJ (2019). Reduced sphingolipid hydrolase activities, substrate accumulation and ganglioside decline in Parkinson’s disease. Mol Neurodegener..

[69] Woeste MA, Stern S, Raju DN, Grahn E, Dittmann D, Gutbrod K (2019). Species-specific differences in nonlysosomal glucosylceramidase GBA2 function underlie locomotor dysfunction arising from loss-of-function mutations. J Biol Chem.

[70] Gatchel J, Watase K, Thaller C, Carson J, Jafar-Nejad P (2008). The insulin-like growth factor pathway is altered in spinocerebellar ataxia type 1 and type 7. Proc Natl Acad Sci USA.

[71] Ingram M, Wozniak EAL, Duvick L, Yang R, Bergmann P, Carson R (2016). Cerebellar transcriptome profiles of ATXN1 transgenic mice reveal SCA1 disease progression and protection pathways. Neuron.

[72] Serra H, Byam C, Lande J, Tousey S, Zoghbi H (2004). Gene profiling links SCA1 pathophysiology to glutamate signaling in Purkinje cells of transgenic mice. Hum Mol Genet.

[73] Driessen TM, Lee PJ, Lim J (2018). Molecular pathway analysis towards understanding tissue vulnerability in spinocerebellar ataxia type 1. Elife.

[74] Napierala JS, Li Y, Lu Y, Lin K, Hauser LA, Lynch DR (2017). Comprehensive analysis of gene expression patterns in Friedreich’s ataxia fibroblasts by RNA sequencing reveals altered levels of protein synthesis factors and solute carriers. DMM Dis Model Mech.

[75] Toonen LJA, Overzier M, Evers MM, Leon LG, Van Der Zeeuw SAJ, Mei H (2018). Transcriptional profiling and biomarker identification reveal tissue specific effects of expanded ataxin-3 in a spinocerebellar ataxia type 3 mouse model. Mol Neurodegener.

[76] Gerstner N, Kehl T, Lenhof K, Müller A, Mayer C, Eckhart L (2020). GeneTrail 3: advanced high-throughput enrichment analysis. Nucleic Acids Res.

[77] Morani F, Doccini S, Sirica R, Paterno M, Pezzini F, Ricca I (2019). Functional transcriptome analysis in ARSACS KO cell model reveals a role of sacsin in autophagy. Sci Rep.

[78] Esteves AR, Cardoso SM (2020). Differential protein expression in diverse brain areas of Parkinson’s and Alzheimer’s disease patients. Sci Rep.

[79] Chappell S, Patel T, Guetta-Baranes T, Sang F, Francis PT, Morgan K (2018). Observations of extensive gene expression differences in the cerebellum and potential relevance to Alzheimer’s disease. BMC Res Notes.

[80] Houck AL, Seddighi S, Driver JA (2018). At the crossroads between neurodegeneration and cancer: a review of overlapping biology and its implications. Curr Aging Sci..

[81] Kanehisa M, Sato Y, Kawashima M, Furumichi M, Tanabe M (2016). KEGG as a reference resource for gene and protein annotation. Nucleic Acids Res.

[82] Hammond TR, Marsh SE, Stevens B (2019). Immune signaling in neurodegeneration. Immunity.

[83] De Rivero Vaccari JP, Brand FJ, Sedaghat C, Mash DC, Dietrich WD, Keane RW (2014). RIG-1 receptor expression in the pathology of Alzheimer’s disease. J Neuroinflamm..

[84] Nachun D, Gao F, Isaacs C, Strawser C, Yang Z, Dokuru D (2018). Peripheral blood gene expression reveals an inflammatory transcriptomic signature in Friedreich’s ataxia patients. Hum Mol Genet.

[85] Delatycki MB, Bidichandani SI (2019). Friedreich ataxia—pathogenesis and implications for therapies. Neurobiol Dis..

[86] Liu J, Wang F (2017). Role of neuroinflammation in amyotrophic lateral sclerosis: cellular mechanisms and therapeutic implications. Front Immunol.

[87] Hooten KG, Beers DR, Zhao W, Appel SH (2015). Protective and toxic neuroinflammation in amyotrophic lateral sclerosis. Neurotherapeutics.

[88] Saudubray JM, Garcia-Cazorla A (2018). An overview of inborn errors of metabolism affecting the brain: from neurodevelopment to neurodegenerative disorders. Dialogues Clin Neurosci..

[89] Dulce P-G, Christophe M, Minh Bao H, Fernando S, Ludmilla S, Diaz Julia Elisa S (2011). Glycosaminoglycans, protein aggregation and neurodegeneration. Curr Protein Pept Sci..

[90] Wilkinson K, El Khoury J (2012). Microglial scavenger receptors and their roles in the pathogenesis of Alzheimer’s disease. Int J Alzheimer’s Dis..

[91] Husemann J, Silverstein SC (2001). Expression of scavenger receptor class B, type I, by astrocytes and vascular smooth muscle cells in normal adult mouse and human brain and in Alzheimer’s disease brain. Am J Pathol.

[92] Tesseur I, Zou K, Esposito L, Bard F, Berber E, Van Can J (2006). Deficiency in neuronal TGF-β signaling promotes neurodegeneration and Alzheimer’s pathology. J Clin Invest.

[93] Estrada LD, Oliveira-Cruz L, Cabrera D (2018). Transforming growth factor beta type I role in neurodegeneration: implications for Alzheimer’s disease. Curr Protein Pept Sci.

[94] Xu F, Na L, Li Y, Chen L (2020). Roles of the PI3K/AKT/mTOR signalling pathways in neurodegenerative diseases and tumours. Cell Biosci.

[95] Liu Q, Qiu J, Liang M, Golinski J, Van Leyen K, Jung JE (2014). Akt and mTOR mediate programmed necrosis in neurons. Cell Death Dis.

[96] Sánchez-Alegría K, Flores-León M, Avila-Muñoz E, Rodríguez-Corona N, Arias C (2018). PI3K signaling in neurons: a central node for the control of multiple functions. Int J Mol Sci..

[97] Ribeiro M, Rosenstock TR, Oliveira AM, Oliveira CR, Rego AC (2014). Insulin and IGF-1 improve mitochondrial function in a PI-3K/Akt-dependent manner and reduce mitochondrial generation of reactive oxygen species in Huntington’s disease knock-in striatal cells. Free Radic Biol Med.

[98] Toonen LJA, Overzier M, Evers MM, Leon LG, Van Der Zeeuw SAJ, Mei H (2018). Transcriptional profiling and biomarker identification reveal tissue specific effects of expanded ataxin-3 in a spinocerebellar ataxia type 3 mouse model. Mol Neurodegener.

[99] Palmer MJ, Mahajan VS, Trajman LC, Irvine DJ, Lauffenburger DA, Chen J (2008). Interleukin-7 receptor signaling network: An integrated systems perspective. Cell Mol Immunol..

[100] Kang J, Coles M (2012). IL-7: the global builder of the innate lymphoid network and beyond, one niche at a time. Semin Immunol Semin Immunol.

[101] Mkhikian H, Grigorian A, Li CF, Chen HL, Newton B, Zhou RW (2011). Genetics and the environment converge to dysregulate N-glycosylation in multiple sclerosis. Nat Commun.

[102] Gregory SG, Schmidt S, Seth P, Oksenberg JR, Hart J, Prokop A (2007). Interleukin 7 receptor α chain (IL7R) shows allelic and functional association with multiple sclerosis. Nat Genet.

[103] Li WQ, Jiang Q, Khaled AR, Keller JR, Durum SK (2004). Interleukin-7 inactivates the pro-apoptotic protein bad promoting T cell survival. J Biol Chem.

[104] Zheng Q, Huang T, Zhang L, Zhou Y, Luo H, Xu H (2016). Dysregulation of ubiquitin-proteasome system in neurodegenerative diseases. Front Aging Neurosci.

[105] Thomas MH, Pelleieux S, Vitale N, Olivier JL. Arachidonic acid in Alzheimer’s disease. J Neurol Neuromed. 2016;1. www.jneurology.com. Accessed 27 Mar 2021.

[106] Tang KS (2014). Protective effect of arachidonic acid and linoleic acid on 1-methyl-4-phenylpyridinium-induced toxicity in PC12 cells. Lipids Health Dis.

[107] Iljina M, Tosatto L, Choi ML, Sang JC, Ye Y, Hughes CD (2016). Arachidonic acid mediates the formation of abundant alpha-helical multimers of alpha-synuclein. Sci Rep.

[108] Di Gregorio E, Borroni B, Giorgio E, Lacerenza D, Ferrero M, Lo Buono N (2014). ELOVL5 mutations cause spinocerebellar ataxia 38. Am J Hum Genet.

[109] Hanna VS, Hafez EAA (2018). Synopsis of arachidonic acid metabolism: a review. J Adv Res.

[110] Angelova PR, Esteras N, Abramov AY (2021). Mitochondria and lipid peroxidation in the mechanism of neurodegeneration: finding ways for prevention. Med Res Rev.

[111] Gasperi V, Sibilano M, Savini I, Catani MV. Niacin in the central nervous system: an update of biological aspects and clinical applications. Int J Mol Sci. 2019; 20. /pmc/articles/PMC6412771/. 10.3390/ijms20040974 PMC6412771 30813414

[112] Fricker RA, Green EL, Jenkins SI, Griffin SM. The influence of nicotinamide on health and disease in the central nervous system. Int J Tryptophan Res. 2018;11. /pmc/articles/PMC5966847/. 10.1177/1178646918776658 PMC5966847 29844677

[113] Balarabe SA, Watila MM (2015). Role of vitamins and cofactors in the management of oxidative stress-induced neuromuscular diseases. NJBAS.

[114] Worgall TS (2008). Regulation of lipid metabolism by sphingolipids. Lipids Health Dis.

[115] Liu JP, Tang Y, Zhou S, Toh BH, McLean C, Li H (2010). Cholesterol involvement in the pathogenesis of neurodegenerative diseases. Mol Cell Neurosci..

[116] Petrov AM, Kasimov MR, Zefirov AL (2016). Brain cholesterol metabolism and its defects: linkage to neurodegenerative diseases and synaptic dysfunction. Acta Nat..

[117] Abdel-Khalik J, Yutuc E, Crick PJ, Gustafsson JÅ, Warner M, Roman G (2017). Defective cholesterol metabolism in amyotrophic lateral sclerosis. J Lipid Res.

[118] Nóbrega C, Mendonça L, Marcelo A, Lamazière A, Tomé S, Despres G (2019). Restoring brain cholesterol turnover improves autophagy and has therapeutic potential in mouse models of spinocerebellar ataxia. Acta Neuropathol.

[119] McLoughlin HS, Moore LR, Paulson HL (2020). Pathogenesis of SCA3 and implications for other polyglutamine diseases. Neurobiol Dis..

[120] Martin LA, Kennedy BE, Karten B (2016). Mitochondrial cholesterol: mechanisms of import and effects on mitochondrial function. J Bioenerg Biomembr..

[121] Kim EK, Choi EJ (2010). Pathological roles of MAPK signaling pathways in human diseases. Biochim Biophys Acta Mol Basis Dis..

[122] Oluwaseun Fadaka A, Adeleke Ojo O, Adetutu Osukoya O, Akuboh O, Ajiboye BO (2017). Role of p38 MAPK signaling in neurodegenerative diseases: a mechanistic perspective. Ann Neurodegener Disord..

