# Supplementary material for: Transcriptomic characterization of tissues from patients and subsequent pathway analyses reveal biological pathways that are implicated in spastic ataxia
Source: Cell Biosci. 2022 Mar 11;12:29. doi: 10.1186/s13578-022-00754-1 (PMC8917697; doi:10.1186/s13578-022-00754-1)
Supplement: Supplementary file 1 — Additional file 1: Figure S1. A) Bright-field microscopy of CTRL DANs at 45 days of differentiation showing typical morphology with elongated and branching processes B) ICC of the pan-neuronal marker TUJ1 expressed by CTRL DANs at 35 days of differentiation C) ICC showing expression of the neuronal (TUJ1) and dopaminergic (TH) markers in DANs cultures at 50 days of differentiation from one control and one patient’s line. Most neuronal cells exhibit co-expression of TUJ1 and TH, demonstrating their dopaminergic identity. Abbreviations: CTRL: control, DANs: dopaminergic neurons, TH: tyrosine hydroxylase, TUBB3: beta-III tubulin. [file 13578_2022_754_MOESM1_ESM.docx]

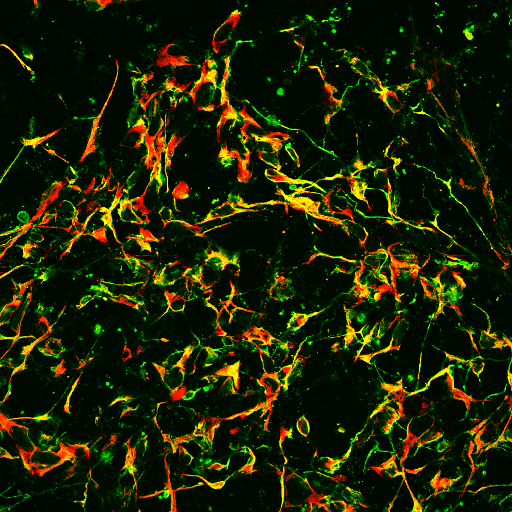





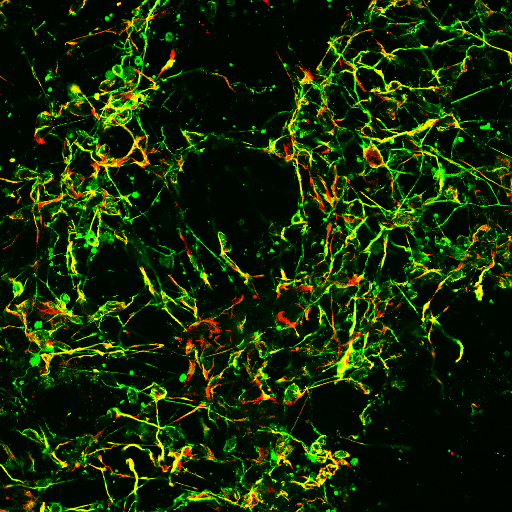



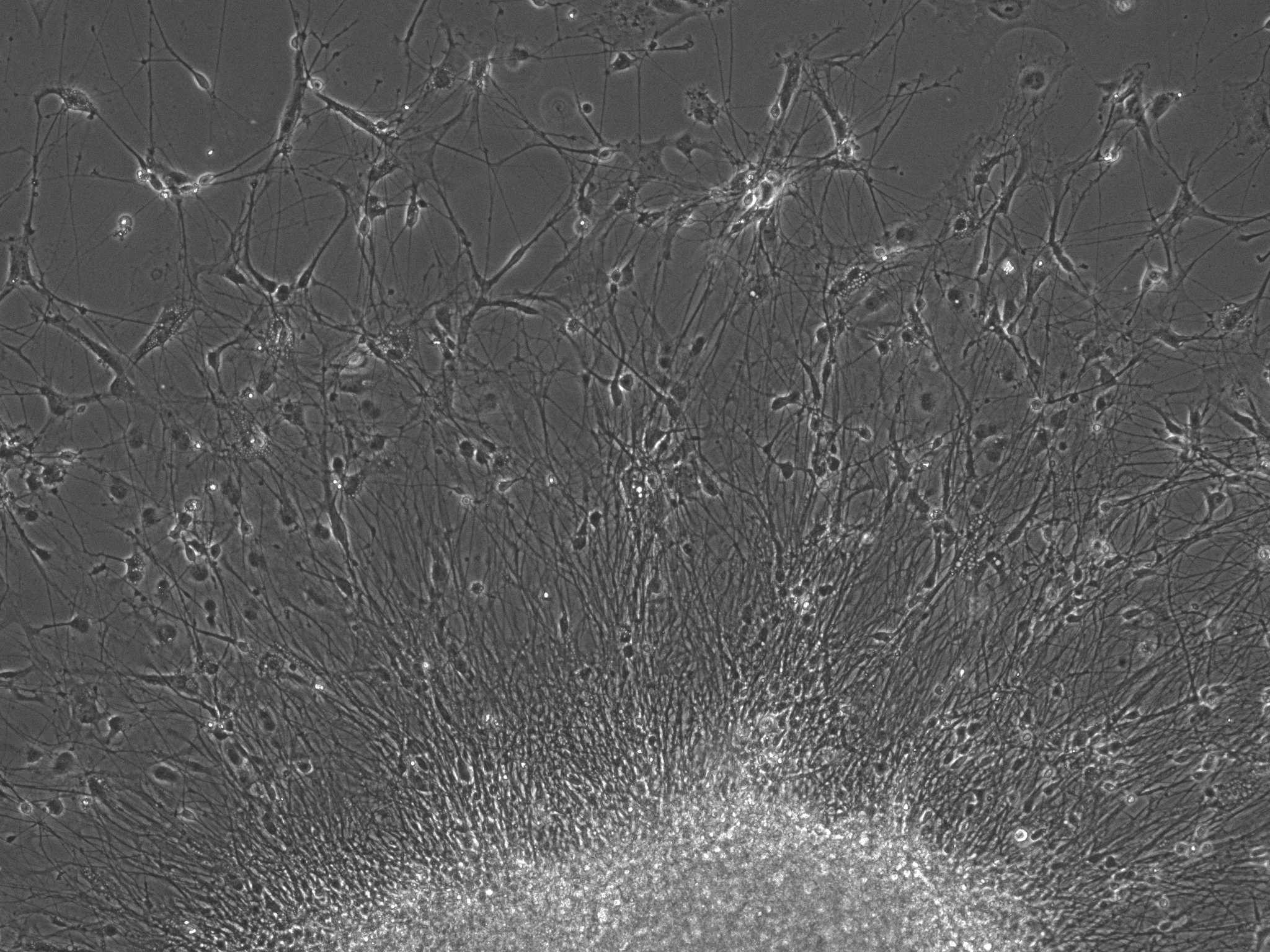

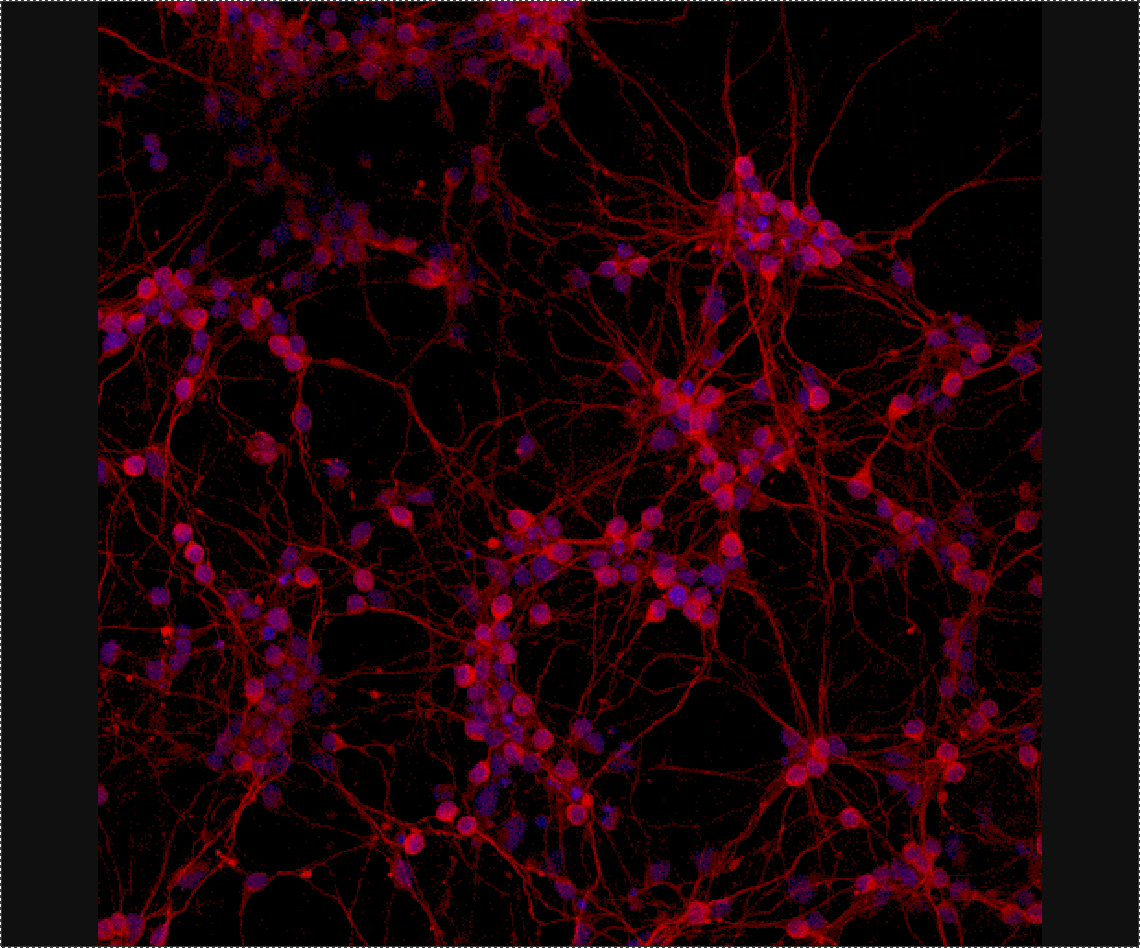


A

B

C

PATIENT

CTRL

TH

TUBB3/TH

TUBB3

TUBB3/DAPI

***Figure S1*** A) Bright-field microscopy of CTRL DANs at 45 days of differentiation showing typical morphology with elongated and branching processes B) ICC of the pan-neuronal marker TUJ1 expressed by CTRL DANs at 35 days of differentiation C) ICC showing expression of the neuronal (TUJ1) and dopaminergic (TH) markers in DANs cultures at 50 days of differentiation from one control and one patient’s line. Most neuronal cells exhibit co-expression of TUJ1 and TH, demonstrating their dopaminergic identity. Abbreviations: CTRL: control, DANs: dopaminergic neurons, TH: tyrosine hydroxylase, TUBB3: beta-III tubulin
